# Not only as a Cullin3 ligase adaptor, SPOP may also act as a molecular bridge

**DOI:** 10.1016/j.gendis.2026.102189

**Published:** 2026-04-14

**Authors:** Yidong Ge, Lili Kong, Yuxuan Li, Xiaofeng Jin

**Affiliations:** Department of Biochemistry and Molecular Biology, Health Science Center, Ningbo University, Ningbo, Zhejiang 315211, China

**Keywords:** Cancer, Molecular bridge, Platform, SPOP, Ubiquitination

## Abstract

Speckle-type POZ protein (SPOP), a substrate-binding adaptor of the Cullin3/RING-box1 E3 ubiquitin ligase complex, induces degradative or non-degradative ubiquitination of substrates, playing a vital role in various signaling pathways, including androgen receptor (AR)/estrogen receptor (ER)-associated signal transduction, DNA damage response, and immunity. A mountain of evidence shows that cancer-associated SPOP mutations or abnormal expression of SPOP exhibit carcinogenic or tumor-suppressive properties in human diseases, such as prostate cancer or kidney cancer. In this review, we not only summarize the structure, regulation, and pathological role of SPOP, but also highlight the SPOP-interacting proteins and their structural characteristics. Based on this, we propose a novel model in which SPOP may act as a molecular bridge, facilitating interactions between its bound proteins, which provides the possibility of forming several molecular platforms, thus gathering oncoproteins closer, resulting in the appearance of certain carcinogenic characteristics of SPOP.

## Introduction

In 1997, Nagai et al first identified a novel nuclear antigen, the speckle-type POZ protein (SPOP), which was recognized by scleroderma sera and isolated through expression cloning from HeLa cell cDNA libraries.^1^ They found that SPOP appeared as a coarse speckle-like pattern in the nucleus, and that it shared 62.7% identity with the putative protein T16H12.5 in *C. elegans*, and further that its NS domain shared 91% identity with T16H12.5.^1^ In 2001, Zapata et al have identified that the N-terminal of SPOP contains a tumor necrosis factor-receptor associated factor (TRAF) domain, which interacts with TRAF2 and TRAF6, but does not exhibit TRAF-mediated nuclear factor kappa B (NF-κB) inhibition.^2^ Although Takahashi et al discovered an interaction between SPOP and MacroH2A1 in 2002, they did not identify its E3 ligase activity.^3^ It was not until 2005 that Hernandez-Munoz et al first described an E3 ligase consisting of SPOP and Cullin3, which mediates the ubiquitination of the PcG protein BMI1 and MacroH2A1 in a non-degradative manner, which appeared on the cover of *Proceedings of the National Academy of Sciences*.^4^ In 2012, Barbieri et al first sequenced 112 pairs of normal prostate and prostate cancer (PCa) tissues and found that SPOP mutations occurred at a frequency of 6%–15% in localized and advanced PCa, and pointed out that mutations at Y87, W131, and F133 disrupted the interaction between SPOP and substrates, thus promoting the development of PCa.^5^ At the same time, Le Gallo et al have revealed that SPOP is frequently mutated in endometrial cancer (EC), with a mutation frequency of up to 8%, and its mutation sites are mainly located in the MATH domain.^6^ Spurred by these findings, numerous studies have been conducted to unveil the roles of SPOP in physiological and pathological processes (Fig. 1A).

**Figure 1:**
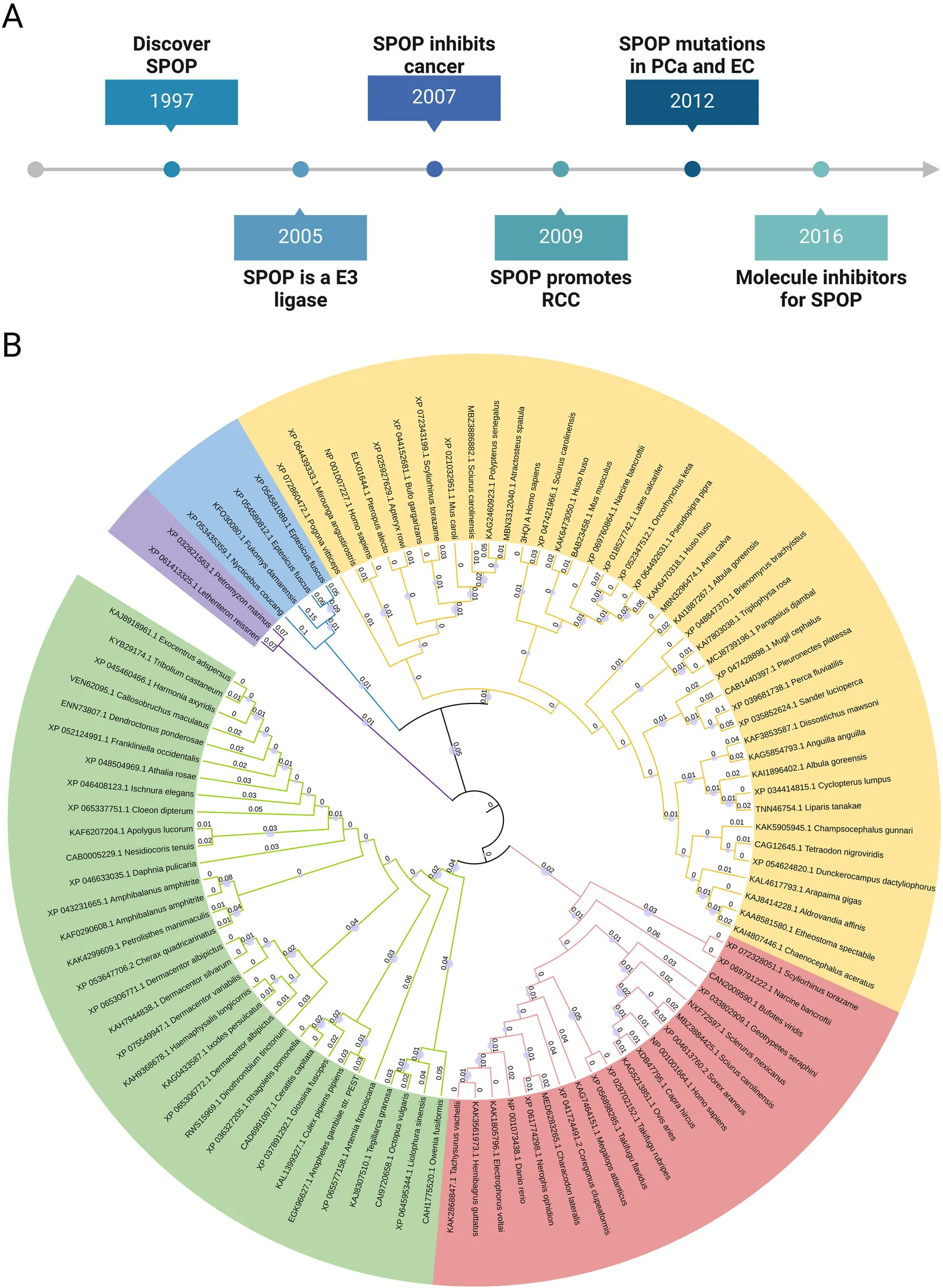
The development of SPOP. **(A)** SPOP was first discovered in 1997, and in the following years, the functions of SPOP and its small molecule inhibitors have been gradually discovered. **(B)** Phylogenetic analysis reveals deep evolutionary conservation of SPOP. A phylogenetic tree was constructed using 100 SPOP homologous protein sequences identified across 85 different species. The analysis demonstrates that SPOP is a highly conserved protein throughout evolution. Notably, human SPOP exhibits a high degree of amino acid identity with its ortholog in *Mirounga angustirostris*, underscoring strong evolutionary pressure and functional constraint on this protein.

In nature, there are 100 homologous SPOP proteins, which are distributed across 85 different species (Fig. 1B). Among them, the Speckle-type POZ protein in *Homo sapiens* was 100% identical to the hypothetical protein GH733_014152 in *Mirounga leonine*, speckle-type POZ protein, isoform CRA_b in *Mus musculus*, and the speckle-type POZ protein in *Bos mutus*, which suggests that SPOP is a relatively conserved protein in the process of evolution (Fig. 1B). In chickens and zebrafish, the immune and inflammatory responses mediated by the conserved SPOP can resist the threats of bacteria and viruses.^7^, ^8^, ^9^ Cullin3 is one of the most widely studied components of the ubiquitin–proteasome system (UPS), in which SPOP is a pivotal adaptor of Cullin3 interacting with the substrate.^10^ The main function of SPOP is to regulate the stability or localization of its target proteins by intracellular ubiquitination modification.^10^ In fact, the function of SPOP mainly depends on the biological activity of target proteins, including cancer inhibition, cancer promotion, and regulation of organ development.^11^ Basically, SPOP inhibits the occurrence and development of cancers by mediating the ubiquitination and degradation of oncoproteins.^11^ For example, SPOP inhibits the proliferation, metastasis, and invasion of PCa and EC by mediating ubiquitination and degradation of AR and estrogen receptor alpha (ERα), respectively.^12^^,^^13^ In addition, SPOP can bind to some target proteins and change their subcellular localization without undergoing proteolytic processes, such as MacroH2A and inverted formin-2 (INF2).^4^^,^^14^ In recent years, it has been gradually discovered that SPOP has its cancer-promoting side, not only in kidney cancer, ovarian cancer, and cervical cancer, as well as ambiguous role in hepatocellular carcinoma (HCC).^15^, ^16^, ^17^, ^18^

Previous reviews have masterfully cataloged the upstream regulators and downstream substrates of SPOP, providing an invaluable resource for the field.^10^^,^^11^^,^^19^, ^20^, ^21^ However, this wealth of information is often presented in a manner that segments SPOP's functions by individual substrates or pathways. This approach, while foundational, falls short of providing a unified conceptual framework that explains how SPOP integrates these disparate cellular activities and, crucially, why it exhibits paradoxical, context-dependent roles in cancer. A new synthesis is needed to move beyond the “list” and place SPOP's multifaceted activities into an integrative physiological and pathological context. In the present review, we first introduce the structure of SPOP in detail and propose its theoretical basis as a molecular bridge. Then, we describe the regulation of SPOP, mainly regarding the expression and activity of SPOP. In addition, we elucidated the role of SPOP in various diseases, including its effects on cancers, the nervous system, immunity, inflammation, and development.

### The structures of SPOP

SPOP is an E3 ligase adaptor composed of 374 amino acids, which includes 4 distinct functional domains: MATH domain (28–166aa), BTB domain (184–297aa), BACK domain (297–359aa), and NLS domain (363–374aa) (Fig. 2A).^17^ The C-terminal NLS domain plays a decisive role in the localization of SPOP in the nucleus, while the NLS domain-deficient SPOP is mainly located in the cytoplasm.^11^ The non-classical truncation of the BACK domain enables the BACK domain to be dimerized, and its interface is formed by the BACK domain and the BTB domain.^21^ In addition, the C-terminal of the BACK allows SPOP to form oligomers (Fig. 2A), which enhances the affinity between SPOP and the substrate and E2, thus promoting the E3 activity of SPOP.^21^ The BTB domain is responsible for two functions; on the one hand, it contributes to the formation of dimerization of SPOP (Fig. 2A), and on the other hand, it mediates the binding of SPOP to Cullin3.^11^ The MATH domain with an antiparallel β-sandwich structure at the N-terminal is the most important domain for SPOP to exert E3 ligase activity, which selectively recruits and binds to substrates.^22^ Furthermore, the MATH domain is also the binding region of most SPOP-targeting inhibitors, such as SPOP-IN-6B (Fig. 2B).

**Figure 2:**
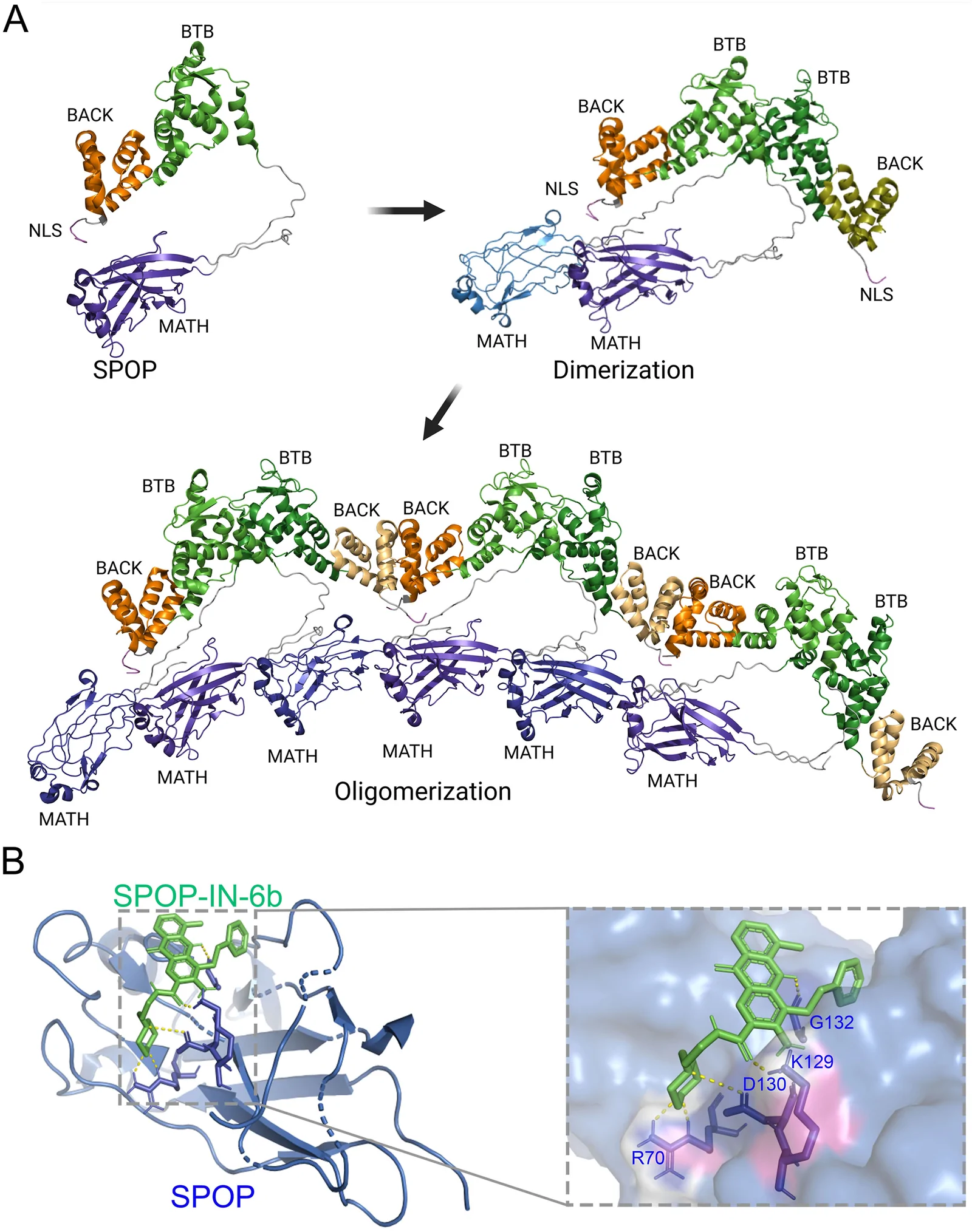
The crystal structure of SPOP. **(A)** SPOP is composed of the MTAH domain, BTB domain, BACK domain, and NLS domain. BTB domain-mediated dimerization and BACK domain-mediated oligomerization are the structural basis of the molecular bridge constructed by SPOP. **(B)** Molecular docking results showed that SPOP-IN-6b, a targeted inhibitor of SPOP, binds to R70, K129, D130, and G132 of SPOP.

SPOP mutations occur primarily as early events in the development of PCa and EC and are concentrated within the MATH domain.^23^ Mutations at Y87, F102, F125, W131, and F133 in the MATH domain occurred mainly in PCa, while mutations at S14, E45, E46, E47, E50, E78, S80, R99, M117, R121, W131, D140, and L282 frequently appear in EC.^11^ SPOP mutations show loss of function and negative dominant function in PCa, while gain of function in EC.^24^ In PCa with SPOP mutations, the expression of AR-regulated transcripts is increased, and the overall survival of these patients improves after hormone therapy.^24^ Whereas in EC, the expression of hormone receptors is dramatically reduced, and the difference in overall survival depends on the hotspot mutation and histological type.^24^ Furthermore, mutation (R221C) in the BTB domain reduces SPOP oligomerization and shifts the size distribution of SPOP oligomers to smaller sizes.^25^ As a result, this mutation reduced its binding to the substrate death domain-associated protein 6 (Daxx), but unexpectedly enhanced its polyubiquitination of Daxx due to the enhanced liquid–liquid phase separation.^25^ The SPOP mutants Y353E and R354H in the BACK domain can block the dimerization of the BACK domain.^25^ SPOP-Y353E blocks BACK domain dimerization, which markedly inhibited the ubiquitination of Daxx, contributing to Daxx mislocalization.^25^ However, SPOP-R354H results in a decrease of BACK domain dimerization and the size of SPOP oligomers, and an increase in ubiquitination of substrates.^25^ Therefore, mass spectrum-based targeted proteomics, which enables the multi-isomer specific detection of mutated SPOP proteins in cell lysates, offers great potential for the development of biomarkers for PCa and EC.^26^

In general, SPOP-binding substrates have one or more specific SPOP-binding consensus (SBC) motifs, which are usually composed of five amino acid residues, Φ-π-S-S/T-S/T (Φ: non-polar amino acid, π: polar amino acid).^11^ Most of the SBC motifs of substrates are bound to the MATH domain of SPOP, which is dependent on polar and hydrophobic interactions.^21^ Given that the affinity of a single SBC motif for SPOP is relatively weak, substrates typically contain multiple SBC motifs. The presence of multiple motifs enhances their cooperative binding to SPOP.^27^ Additionally, the substrate can trigger liquid–liquid phase separation of SPOP and co-localization between SPOP membraneless organelles.^28^ However, disease-associated SPOP mutations lead to the accumulation of oncogenic proteins and interfere with liquid–liquid phase separation and co-localization of membraneless organelles, suggesting that substrate-directed phase separation of this E3 ligase is the basis for ubiquitin-dependent protein homeostasis regulation.^28^

Emerging evidence reveals that the oligomerization landscape of SPOP is more complex than previously appreciated.^29^ A recent structural study proposes a dynamic equilibrium between two massive quaternary assemblies that fundamentally regulates SPOP activity.^29^ In this model, an auto-inhibited, ring-like “double-donut” assembly, which can exceed 2 MDa in size, sterically hinders substrate access by burying the MATH domains at the inter-donut interface.^29^ This state exists in equilibrium with the active, linear filamentous form.^29^ Critically, this equilibrium is susceptible to perturbation by cancer-associated mutations. For instance, gain-of-function mutations prevalent in EC (*e.g.*, E47K) destabilize the double-donut, favoring the active filament.^29^ Conversely, certain mutations, including some found in PCa (*e.g.*, K134N), appear to promote inactivation not only by impairing the substrate-binding groove but also by stabilizing the auto-inhibited double-donut.^29^ This mechanism, governed by large-scale conformational switching, provides a compelling structural rationale for the context-dependent oncogenic roles of SPOP and highlights oligomerization as a central regulatory node.^29^

### The regulation of SPOP

#### The promoting effects

##### The increased expression of SPOP

Increased SPOP expression encompasses both elevated SPOP mRNA levels and stabilization of the SPOP protein, leading to its hyperactivation (Fig. 3; Table S1).

**Figure 3:**
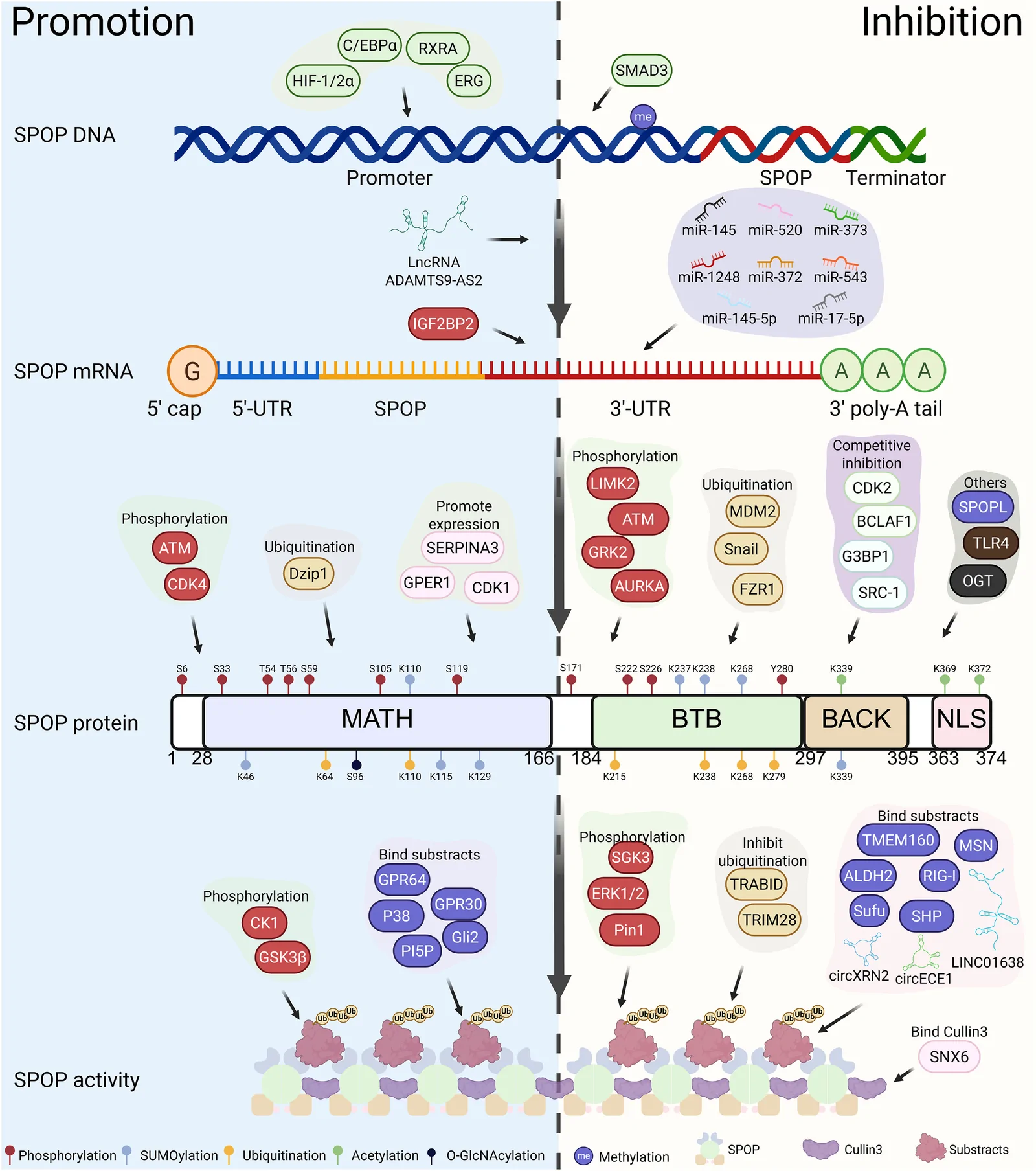
Regulatory network governing SPOP expression and activity. This schematic diagram illustrates the multi-level regulation of SPOP, from gene transcription to post-translational modification of the mature protein. The model summarizes key upstream regulators that either enhance or suppress SPOP's function. Notably, regulators distributed in the left half of the figure, against a blue background, represent pathways that promote SPOP expression or activity. These include transcriptional activators (*e.g.*, C/EBPα, HIF-1α), mRNA stabilizers (*e.g.*, IGF2BP2), and post-translational modifications that prevent its degradation (*e.g.*, CDK4-mediated phosphorylation). Conversely, factors located in the right half, against a yellow background, represent inhibitory mechanisms. These encompass transcriptional repression (*e.g.*, promoter hypermethylation), mRNA targeting by miRNAs, and post-translational modifications that trigger SPOP degradation (*e.g.*, LIMK2-mediated phosphorylation). This intricate network underscores the precise control over SPOP's tumor-suppressive or oncogenic functions in different cellular contexts.

First, the primary mechanism by which SPOP mRNA is up-regulated is the enhancement of *SPOP* transcription, which is regulated by transcription factors that bind to the *SPOP* promoter. Yao et al found that C/EBPα bound to −1144 to −1135, −815 to −806, and +55 to +64 on the *SPOP* promoter, thereby promoting SPOP transcription.^30^ On the contrary, hypermethylation of CpG islands of the *SPOP* promoter leads to the elimination of C/EBPα binding, thereby inhibiting SPOP transcription.^30^ Similarly, the transcription factors ETS-related gene (ERG), hypoxia-inducible factor-1α (HIF-1α), and HIF-2α bind to the SPOP promoter, enhancing its transcription and up-regulating SPOP mRNA expression, ultimately leading to the down-regulation of SPOP substrates.^31^, ^32^, ^33^ In addition, lncRNA ADAMTS9-AS2 positively regulates the expression of SPOP mRNA in gastric carcinoma, thereby inhibiting the proliferation of gastric carcinoma and tumorigenicity of stem cells.^34^ However, the underlying mechanism for this up-regulation of SPOP mRNA has not been elucidated. In addition, IGF2BP2 enhanced the stability of SPOP mRNA by recognizing m^6^A-modified SPOP 3′-UTR.^35^

Second, the stabilization of the SPOP protein can also lead to the hyperfunction of SPOP, which is mainly caused by the dysregulation of the UPS that mediates the degradation of SPOP. For instance, cyclin D1–cyclin-dependent kinase 4 (CDK4)-mediated phosphorylation of SPOP S6 disrupts its binding to fizzy-related protein homolog 1 (FZR1) through recruitment of 14-3-3γ, thereby inhibiting FZR1-mediated ubiquitination and degradation of SPOP.^36^ Although DAZ-interacting protein (Dzip1) can also inhibit the degradation of SPOP via the UPS proteasome to stabilize SPOP, the specific E3 ligase that mediates this degradation is unclear.^37^ Additionally, G-protein coupled estrogen receptor 1 (GPER1), cyclin-dependent kinase 1 (CDK1), and SPOP upstream protein serine protease inhibitor clade A member 3 (SERPINA3) can up-regulate the expression of SPOP protein, thereby promoting SPOP-mediated ubiquitination and degradation of ERα, programmed death ligand 1 (PD-L1), and NF-κB, respectively.^38^, ^39^, ^40^ Among them, GPER1 not only increases the expression of SPOP mRNA, but also increases the expression of SPOP protein.^38^

##### The increased activity of SPOP

At present, there are two main ways to enhance the activity of SPOP: phosphorylation of SPOP itself and phosphorylation of SPOP substrates (Fig. 3; Table S1).

The enzyme that mediates the phosphorylation of SPOP is the ataxia-telangiectasia mutated (ATM), which promotes cell cycle progression and DNA damage repair.^41^, ^42^, ^43^ When DNA is damaged, ATM-mediated phosphorylation of SPOP S119 leads to conformational changes in SPOP that prompt SPOP to bind to 53-binding protein 1 (53BP1), which induces the polyubiquitination of 53BP1, removes 53BP1 from chromatin, and promotes homologous recombination repair during DNA replication.^41^ SPOP S119 phosphorylated by ATM also enhances the interaction between SPOP and homeodomain-interacting protein kinase 2 (HIPK2), and induces non-degradative ubiquitination of HIPK2, which enhances the phosphorylation activity of HIPK2 to heterochromatin protein 1 gamma (HP1γ), and further promotes the dissociation of HP1γ from trimethylated histone H3 (H3K9me3), thus initiating DNA damage repair.^42^ In addition, CDK4 stimulates SPOP by phosphorylating SPOP, leading to proteasome degradation of PD-L1 in triple-negative breast cancer cells.^44^

The primary enzyme mediating substrate phosphorylation is casein kinase 1 (CK1), which phosphorylates serine residues within the SBCs of substrates, thereby promoting their SPOP-mediated ubiquitination and degradation.^45^, ^46^, ^47^ For example, CK1 promotes SPOP-mediated ubiquitination and degradation of ASC amino-acid transporter 2 (ASCT2) by phosphorylating SPOP-bound serine residues on ASCT2.^45^ In the same way, CK1 promotes SPOP-mediated ubiquitination and degradation of 3-phosphoinositide-dependent protein kinase-1 (PDK1) and Ewing sarcoma (EWS)–friend leukemia integration 1 (FLI1) by phosphorylating the second serine residue in VSSSSSS of PDK1 and all three serine residues in VTSSS of FLI1, respectively.^46^^,^^47^ Jiang et al also show that glycogen synthase kinase 3β (GSK3β) can phosphorylate the same serine residue in the SBC of PDK1, thereby promoting SPOP-mediated ubiquitination and degradation of PDK1.^47^ However, the promoting effect of GLI family zinc finger protein 2 (Gli2) on SPOP-mediated ubiquitination and degradation of RNA polymerase II binding subunit 3/7 (Rpb3/7) is not through the phosphorylation of the substrate, but through the interaction with the substrate, which has also been demonstrated in drosophila.^48^

Moreover, the interactions between some membrane receptors, such as G-protein coupled receptor 30 (GPR30) and GPR64, and SPOP enhance the activity of SPOP.^49^^,^^50^ The interaction between GPR30 and SPOP promotes SPOP-mediated ubiquitination and degradation of NOD-like receptor protein 3 (NLRP3), thus inhibiting the activation of the inflammasome and causing neuronal pyroptosis in ischemic stroke.^49^ Ceramides produced by sphingomyelin phosphodiesterase 1 (SMPD1) induce cleavage of the intracellular domain of GPR64, which triggers SPOP-mediated degradation of Rap1-interacting factor 1 homolog (RIF1), leading to proliferation of Ewing sarcoma.^50^ In addition, p38 and phosphatidylinositol 5 phosphate (PI5P) stimulate the interaction between SPOP and its substrate in a p38-dependent manner.^51^

### The inhibiting effects

#### The decreased expression of SPOP

The inhibition of SPOP activity was partly reflected in the down-regulation of SPOP expression, including the regulation of SPOP mRNA and protein (Fig. 3; Table S1).

The down-regulation of SPOP mRNA is mainly divided into two parts: inhibition of *SPOP* transcription and blocking of SPOP mRNA translation by miRNAs. Hypermethylation of the *SPOP* promoter leads to reduced binding affinity of retinoid X receptor alpha (RXRA) and C/EBPα to the *SPOP* promoter, resulting in a down-regulation of SPOP expression.^52^^,^^53^ In PCa, when SMAD family member 3 (SMAD3) binds to the binding elements on the *SPOP* promoter, SMAD3 inhibits the expression of SPOP mRNA, resulting in the reduced ubiquitination and degradation of NANOG.^54^ As for miRNAs, the majority of miRNAs bind to the 3′-UTR on the SPOP transcripts, resulting in decreased expression of SPOP, which leads to decreased ubiquitination and degradation of PD-L1, Gli2, etc..^55^^,^^56^ The miRNAs mediating this process include miR-145,^57^ miR-1248,^58^ miR-543,^59^ miR-520,^55^ miR-17-5p,^60^miR-372^61^, miR-373.^61^, and miR-145-5p.^56^ However, miR-873-5p does not directly target the 3′-UTR of SPOP mRNA, but targets heme oxygenase 1 (HMOX1), thus inhibiting the expression of HIF-1α, which inhibits the expression of SPOP.^62^

The down-regulation of SPOP protein is mainly caused by degradation of SPOP via the UPS, which is usually induced by phosphorylation of SPOP. LIM kinase-2 (LIMK2) promotes the phosphorylation of SPOP at S59, S171, and S226, inducing SPOP ubiquitination and degradation, thus suppressing SPOP-mediated ubiquitination and degradation of its substrates, including androgen receptor (AR), androgen receptor splice variant 7 (ARv7), and c-Myc.^63^ Similarly, aurora kinase A (AURKA) also could mediate the phosphorylation of SPOP at S33, T56, and S105, leading to the ubiquitination and degradation of SPOP, which further leads to dysregulation of ubiquitination and degradation of AR, ARv7, and c-Myc.^64^ Furthermore, G-protein coupled receptor kinase 2 (GRK2)-mediated phosphorylation of SPOP at S222 inhibits the dimerization of SPOP and triggers the autoubiquitination and degradation of SPOP.^45^ However, COP9 signalosome complex subunit 6 (CSN6) and Snail promote ubiquitination and degradation of SPOP independently of phosphorylation.^65^^,^^66^ CSN6 promotes ubiquitination and degradation of murine double minute 2 (MDM2)-mediated SPOP, leading to weakened SPOP-mediated ubiquitination and degradation of hydroxymethylglutaryl-CoA synthase 1 (HMGCS1).^65^ Snail binds to the BTB domain of SPOP, thereby promoting ubiquitination and degradation of SPOP.^66^

#### The decreased activity of SPOP

Currently, a variety of upstream suppressors of SPOP have been revealed, which do not affect the expression of SPOP but weaken the binding of SPOP to the substrates, thereby inhibiting SPOP-mediated ubiquitination and degradation (Fig. 3; Table S1). These upstream suppressors exert their functions in two ways: one is to bind to their substrates, and the other is to bind to the MATH domain of SPOP.

### Suppressors bind to substrates

In terms of the number of upstream suppressors, suppressors that compete with SPOP on substrates have been the most extensively studied (Fig. 3; Table S1). For example, aldehyde dehydrogenase 2 (ALDH2) and retinoic acid-inducible gene 1 (RIG-I) bind to the intracellular fragments (260–290 aa) and transmembrane fragments (239–259 aa) of PD-L1, respectively, resulting in weakened binding of SPOP to PD-L1, thereby promoting immune escape and tumor progression in colorectal cancer.^67^^,^^68^ In addition, transmembrane protein 160 (TMEM160) and Moesin (MSN) phosphorylated by Rho-associated protein kinase (ROCK) at T558 competitively bind to PD-L1 with SPOP, thereby inhibiting SPOP-mediated ubiquitination and degradation of PD-L1; however, the specific binding sites of PD-L1 are not revealed.^69^^,^^70^ Similarly, both LINC01638 lncRNA and circECE1 inhibit the interaction between SPOP and c-Myc by binding to 144–240 aa of c-Myc, which contains SBC1 (185-VCSTS-189) of c-Myc, resulting in the stabilization and enhancement of transcriptional activity of c-Myc.^71^^,^^72^ Consistently, circXRN2 inhibits SPOP-mediated ubiquitination and degradation of the large tumor suppressor 1 (LATS1) by binding to 279–525 aa of LATS1, and the SBCs of LATS1, including 327-MQSSS-341 and 429-PQSSS-443, are located in the same fragment where circXRN2 combines with LATS1.^73^ Furthermore, both suppressor of fused homolog (Sufu) and small heterodimer partner (SHP) bind to the substrates of SPOP in the same fragments of the substrates.^74^^,^^75^ Sufu competently binds to the C-terminal (961 aa-end) of Gli2 and the N-terminal (1–477 aa) and C-terminal (1051–1328 aa) of Gli3 with SPOP, and SHP competitively binds to the 301–371 aa of Gli3 with SPOP.^74^^,^^75^

At present, there are also some phosphorylases, including peptidyl-prolyl isomerase (Pin1), extracellular signal-regulated kinase 1/2 (ERK1/2), epidermal growth factor (EGF)/mitogen-activated protein kinase (MAPK)/ERK kinase (MEK)/ERK pathway, and serum and glucocorticoid inducible kinase 3 (SGK3), which mediate the phosphorylation of substrates, disrupting the interaction between SPOP and substrates.^76^, ^77^, ^78^, ^79^ Among them, Pin1 and EGF/MEK/ERK pathway-mediated substrate phosphorylation sites do not occur in SBCs, while ERK1/2 and SGK3 are inside SBCs.^76^, ^77^, ^78^, ^79^ Pin1 impairs the interaction between SPOP and Nanog by mediating the phosphorylation of Nanog at S65 and S71, while the SBC of Nanog is 66-PDSST-70, located in the middle of the phosphorylation sites.^76^ Analogously, the EGF/MEK/ERK pathway blocks SPOP-mediated interleukin enhancer-binding factor 3 (ILF3) ubiquitination and degradation through phosphorylating ILF3 S382, which does not occur in the SBC (360-PPSTT-364).^79^ In contrast, SGK3-mediated phosphorylation of 17beta-hydroxysteroid dehydrogenase type 4 (17βHSD4) S318, located in the SBC (315-RATST-319), leads to the weakened SPOP-mediated non-degradative ubiquitination of 17βHSD4, thereby triggering the degradation of 17βHSD4.^78^ Fan et al found that prostate leucine zipper (PrLZ) could be ubiquitinated and degraded by SPOP binding to 30–42 aa and 46–224 aa of PrLZ.^77^ However, ERK1/2 blocks the interaction between SPOP and PrLZ by mediating phosphorylation of PrLZ at Ser40.^77^ Moreover, they found that interleukin (IL)-6 may regulate PrLZ degradation by promoting phosphorylation of PrLZ, resulting in impaired recognition by SPOP.^77^

Finally, the way tripartite motif-containing protein 28 (TRIM28) and TRAF-binding domain-containing protein (TRABID) inhibit SPOP is not by inhibiting the binding of SPOP to the substrates, but by antagonizing the SPOP-mediated ubiquitination of the substrates.^80^^,^^81^ After combining with TRIM24, TRIM28 prevents SPOP from ubiquitinating TRIM24 without preventing SPOP from binding to TRIM24, thereby promoting the expression of TRIM24.^80^ As for deubiquitinating enzyme TRABID, it promotes the deubiquitination of the ubiquitinated 53BP1 mediated by SPOP, which prevents 53BP1 from dissociating from DNA double-strand breaks, thereby inducing non-homologous end ligations instead of homologous recombination, resulting in chromosomal instability.^81^

### Suppressors bind to SPOP

The suppressors that bind to SPOP either competitively bind to the substrates with SPOP or alter the localization of SPOP, thereby inhibiting its activity (Fig. 3; Table S1).

The suppressors of SPOP that competitively bind to the substrates interact with the MATH domain of SPOP, which is responsible for binding to substrates. Both steroid receptor coactivator-1 (SRC-1) and B-cell lymphoma-2-associated transcription factor 1 (BCLAF1) can competitively bind to the MATH domain of SPOP with PD-L1, thereby inhibiting SPOP-mediated ubiquitination and degradation of PD-L1, leading to immune escape in colorectal cancer and liver cancer, respectively.^82^^,^^83^ Ras-GTPase-activating protein binding protein 1 (G3BP1) can also competitively bind to the MATH domain of SPOP with substrates, thereby weakening the degradation of AR and ERα, leading to the development of PCa and EC, resulting in resistance to enzalutamide and fluvestrant.^12^^,^^13^ Additionally, CDK2 competitively binds to the MATH domain of SPOP with cyclin E1, thereby inhibiting SPOP-mediated polyubiquitination and degradation of cyclin E1.^84^ Similarly, IGF2BP3 competitively binds to SPOP with c-Myc to inhibit the activity of SPOP-mediated degradation of c-Myc.^85^ Utilizing this mechanism, Wang et al and Dong et al discovered two small molecular inhibitors of SPOP (TAT38 and compound 6lc, respectively).^86^^,^^87^ TAT38 and compound 6lc both inhibit the binding of substrates to SPOP through the interaction with the MATH domain, leading to the stability of phosphatase and tensin homolog deleted on chromosome ten (PTEN).^86^^,^^87^ ATM-mediated phosphorylation of SPOP S119, which is also inside the MATH domain, causes SPOP to fail to mediate ubiquitination and degradation of minichromosome maintenance complex component 3 (MCM3), thereby inducing the formation of the MCM3–MCM5 complex, which promotes DNA repair and tolerance to ionizing radiation.^88^

In addition, the suppressors can inhibit SPOP activity by interacting with SPOP, which alters the localization of SPOP.^89^^,^^90^ In most cases, SPOP is mainly localized in the nucleus, but when toll-like receptor 4 (TLR4) is activated, SPOP is recruited to myeloid differentiation primary response 88 (MyD88) and promotes SPOP translocation from the nucleus to the cytoplasm, thereby disrupting the self-association of MyD88 without affecting its ubiquitination and degradation.^89^ However, there is a study showing that SPOP is localized primarily in the cytoplasm in liver cells and mediates the ubiquitination and degradation of the neurite outgrowth inhibitor-B (Nogo-B).^90^ When liver cells evolve into liver cancer cells, SPOP is highly O-GlcNAcylated by O-GlcNAc transferase at S96, which increases the nuclear localization of SPOP and reduces the degradation of Nogo-B.^90^

### Others

In addition, several other mechanisms, as well as some findings, have not been fully studied. One novel mechanism is that sorting nexin-6 (SNX6) reduces the interaction between SPOP and Cullin3 by binding to SPOP, thereby down-regulating Cullin3-mediated PD-L1 ubiquitination and modulating the immune response.^91^ The other is that speckle-type POZ protein-like (SPOPL) may bind to SPOP to form a miniature molecular inhibitor that regulates SPOP-mediated ubiquitination and degradation.^92^

### The effects of SPOP in diseases

#### Updates on the inhibiting effects on cancers

In most cancers, SPOP acts as a tumor suppressor by mediating ubiquitination and degradation of substrate oncoproteins in the development of cancers (Fig. 4; Table S2). Given that our previous review has elaborated on SPOP-mediated ubiquitination and degradation before 2023,^11^ we will not repeat the relevant contents here. However, several SPOP interaction proteins have been identified in the last two years; therefore, we will briefly summarize these updates (Fig. 4A; Table S2).

**Figure 4:**
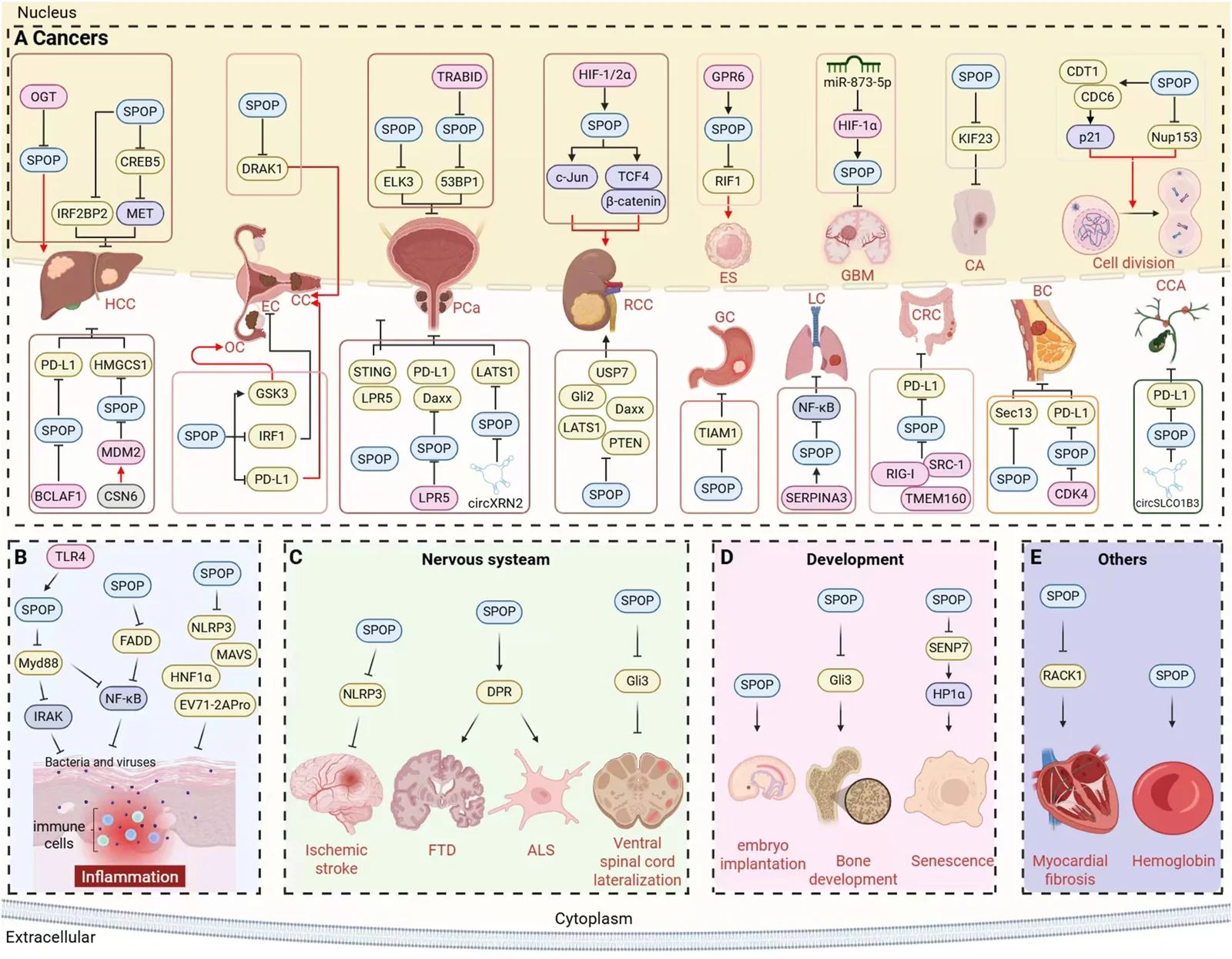
The multifaceted roles of SPOP in human diseases through its E3 ubiquitin ligase activity. **(A)** Context-dependent roles of SPOP in cancers. This panel summarizes the specific signaling pathways and substrates through which SPOP influences various cancer types. The subcellular localization (nucleus or cytoplasm) of key molecular events is indicated. To clearly denote the net oncogenic effect of SPOP in each cancer type, the final arrow pointing directly to the cancer box is color-coded: a red arrow signifies an overall tumor-promoting role, while a black blocked arrow signifies an overall tumor-suppressing role. BC, bladder cancer; BCa, breast cancer; CCA, cholangiocarcinoma; CC, cervical cancer; CRC, colorectal cancer; EC, endometrial cancer; ES, Ewing sarcoma; GC, gastric cancer; GBM, glioblastoma; HCC, hepatocellular carcinoma; LC, lung cancer; OC, ovarian cancer; PCa, prostate cancer; RCC, renal clear cell carcinoma. **(B)** SPOP in immune and inflammatory responses. This panel illustrates the regulatory functions of SPOP in pathways such as the TLR4/MyD88/NF-κB axis, highlighting its role in modulating the production of pro-inflammatory cytokines and the immune response to bacterial and viral infections. **(C)** Neurological functions of SPOP. Depicted here are the mechanisms by which SPOP influences nervous system diseases. **(D)** SPOP in developmental processes. This section outlines the involvement of SPOP in key developmental events. **(E)** Other pathological roles of SPOP. The diagram shows the participation of SPOP in additional pathophysiological conditions, including its impact on myocardial fibrosis and hemoglobin.

In the past two years, the most frequently reported cancers involving SPOP have been those of the digestive system, including HCC,^65^^,^^82^^,^^93^^,^^94^ colorectal cancer,^68^^,^^70^^,^^83^ gastric carcinoma,^95^ cholangiocarcinoma,^96^ and pancreatic cancer.^97^ In HCC, four SPOP substrate proteins, including interferon regulatory factor 2-binding protein 2 (IRF2BP2), cyclic AMP-responsive element-binding protein 5 (CREB5), tumor necrosis factor receptor-associated factor-6 (TRAF6), and HMGCS1, and two upstream suppressors, including BCLAF1 and CSN6, were identified.^65^^,^^82^^,^^93^^,^^94^^,^^98^ SPOP-mediated ubiquitination and degradation of IRF2BP2, TRAF6, and HMGCS1, and SPOP-mediated non-degradation ubiquitination of CREB5, all suppress the occurrence and development of HCC.^65^^,^^93^^,^^94^^,^^98^ However, inhibition of SPOP function by BCLAF1 and SPOP degradation by MDM2, promoted by CSN6, inhibited the degradation of PD-L1 and HMGCS1, respectively, leading to the progression of HCC.^65^^,^^82^ As for colorectal cancer, three upstream suppressors were discovered, namely SRC-1, TMEM160, and RIG-I, which all inhibited SPOP-mediated ubiquitination and degradation of PD-L1, thereby promoting immune escape.^68^^,^^70^^,^^83^ Similarly, circSLCO1B3 increases the expression of PD-L1 by inhibiting SPOP-mediated ubiquitination degradation of PD-L1, thereby contributing to the progression of intrahepatic cholangiocarcinoma.^96^ In gastric carcinoma, SPOP inhibits the cancer progression by mediating ubiquitination and degradation of T-lymphoma invasion and metastasis 1 (TIAM1).^95^ As for pancreatic cancer, it has been found that the overexpression of SPOP recruits more tumor-infiltrating immune cells and improves the survival rate of pancreatic cancer patients, but the relevant interacting proteins are not revealed.^97^

In urinary system cancers, the most updated is PCa, with two studies on bladder cancer.^73^^,^^81^^,^^99^, ^100^, ^101^, ^102^ The newly discovered five substrate proteins in PCa are Jumonji domain-containing 6 (JMJD6), STING, E26 transformation-specific (ETS) transcription factor ELK3 (ELK3), and lipoprotein receptor-related protein 5 (LPR5), all of which can be ubiquitinated and degraded by SPOP, thereby inhibiting PCa development and chemotherapy resistance.^99^, ^100^, ^101^^,^^103^ Among them, LRP5 can also competitively bind to SPOP of Daxx and PD-L1, resulting in decreased degradation of Daxx and PD-L1, further leading to the development of PCa.^99^ As an upstream suppressor of SPOP, TRABID prevents the ubiquitination of 53BP1 by SPOP, inducing non-homologous end ligations, thereby resulting in chromosomal instability of PCa.^81^ In bladder cancer, the newly discovered circXRN2 prevents SPOP-mediated LATS1 degradation, which activates the Hippo signaling pathway to inhibit H3K18 lactylation and lipocalin-2 (LCN2) expression.^73^ Additionally, SPOP-mediated ubiquitination and degradation of signal transducer and activator of transcription 3 (STAT3) inhibit bladder cancer through the STAT3/C–C motif chemokine ligand 2 (CCL2)/IL-6 axis.^102^

In terms of female cancers, recent findings have focused on breast cancer,^44^^,^^104^ EC,^13^^,^^105^ and choriocarcinoma.^106^ The novel substrate identified in breast cancer is Sec13, whose degradation by SPOP weakens the interaction of Sec13 with other proteins in GAP activity towards Rags 2 (GATOR2), thereby decreasing mammalian target of rapamycin (mTOR) activity and inhibiting proliferation and metastasis.^104^ The novel upstream protein is CDK4, which stimulates the proteasome degradation of PD-L1, thereby inhibiting the development of triple-negative breast cancer cells.^44^ In EC, SPOP directly binds to interferon regulatory factor 1 (IRF1), the main transcription factor for PD-L1, which triggers the ubiquitination and degradation of IRF1 to inhibit PD-L1 transcription and the immune escape.^105^ In addition, G3BP1 and IGF2BP3 inhibit SPOP-mediated ubiquitination and degradation, similar to AR in PCa.^13^^,^^85^ As for choriocarcinoma, SPOP inhibits the proliferation, metastasis, and invasion by mediating kinesin family member 23 (KIF23) ubiquitination and degradation.^106^

Besides these cancers, novel SPOP-interacting proteins have been identified in glioblastoma^62^^,^^107^ and lung cancer.^40^ In glioblastoma, miR-873-5p and phosphocreatine inhibit HIF-1α-induced SPOP mRNA expression and SPOP-mediated ubiquitination and degradation of bromodomain-containing protein 2 (BRD2), respectively, resulting in a malignant phenotype and epigenetic reprogramming.^62^^,^^107^ The newly identified SERPINA3 in lung cancer up-regulates SPOP expression, which inhibits NF-κB p65, thereby inhibiting the occurrence and development of cancer.^40^

### The promoting effects on cancers

In 2017, Zhu et al found that SPOP-mediated polyubiquitination of SET domain-containing protein 2 (SETD2) stabilized the SETD2 protein, which promoted trimethylation of histone H3K36 and induced H3K36me3-conjugation alternative splicing events, suggesting that SPOP may act as an oncoprotein.^108^ By 2023, Dai et al showed that SPOP overexpression induced cell proliferation and changed the gene expression profile of normal cells, and that alternative splicing events regulated by SPOP were involved in different cellular processes, including metabolism, environmental information processing, biological systems, *etc*., indicating that SPOP may have a pro-tumor effect.^15^ Although in PCa with SPOP mutant, the expression of AR-regulated transcripts is increased, and the overall survival improves after hormone therapy.^24^ Whereas in EC, the expression of hormone receptors is significantly reduced, and the difference in overall survival depends on the hotspot mutation and histological type, reflecting that SPOP may have different effects in different tissues.^24^ Furthermore, Yang et al identified HS-2, a potential dual-target histone deacetylase 1 (HDAC1)/SPOP candidate compound, which dramatically inhibited tumor growth in nude mice without toxicity, which also demonstrates that the exhibition of SPOP is expected to be used in cancer therapy.^109^

The cancer-promoting effect of SPOP is manifested not only in classical renal clear cell carcinoma (RCC),^18^ but also in HCC,^15^ cervical cancer,^16^ ovarian cancer,^110^ and Ewing sarcoma^50^ revealed in recent years. On the one hand, SPOP promotes the proliferation, migration, and invasion of RCC by mediating the ubiquitination and degradation of PTEN, ubiquitin-specific peptidase 7 (USP7), and LATS1.^18^^,^^111^^,^^112^ On the other hand, SPOP increases the expression of c-Jun and the formation of β-catenin/transcription factor 4 (TCF4) complex, thus triggering the development of RCC.^113^^,^^114^ In addition, HIF-1α and HIF-2α promote the transcription of *SPOP*, which leads to the down-regulation of PTEN, ERK phosphatase, pro-apoptotic molecule Daxx, and Hh pathway transcription factor Gli2, thereby promoting the occurrence of RCC.^31^ Similarly, transcription factor E2 promoter binding factor 1 (E2F1) inhibits the expression of the miR-520/372/373 family, which decreases miR-520/372/373-mediated down-regulation of SPOP mRNA, thereby improving the RCC progression and metastasis.^61^ Several compounds that inhibit SPOP activity have been developed, including E1, TAT38, and 6lc, all of which inhibit SPOP binding to the substrate, thereby stabilizing PTEN and USP7, suggesting that these small molecular inhibitors of SPOP are promising as targeted therapeutics for RCC.^86^^,^^87^^,^^115^

Except for RCC, the overexpression of SPOP promotes the proliferation of normal hepatocytes and changes the gene expression profile and alternative splicing events, participating in cell metabolism, environmental information processing, biological systems, and other related pathways, thus resulting in the occurrence of HCC.^15^ In addition, SPOP is highly O-GlcNAcylated by O-GlcNAc transferase in HCC, which increases the nuclear localization of SPOP and reduces the degradation of Nogo-B, leading to the activation of the Nogo-B/cellular FLICE-like inhibitory protein (c-FLIP) axis and the development of HCC.^90^ As for cervical cancer, SPOP promotes immune escape and paclitaxel resistance by promoting PD-1 away from PD-L1 and the ubiquitination and degradation of death-associated protein kinase-related apoptosis-inducing kinase 1 (DRAK1), respectively.^16^^,^^116^ In ovarian cancer, the expression of SPOP increases, which promotes the migration and invasion of ovarian cancer cells through p-GSK3.^110^ In addition, ceramides produced by SMPD1 induce cleavage of the intracellular domain of GPR64, which triggers SPOP-mediated degradation of RIF1, leading to proliferation of Ewing sarcoma.^50^

### The effects of SPOP on immune and inflammatory

SPOP also plays an important role in immunity and inflammation, mainly in the process of bacterial and viral infections, and the main pathway mediating this modification process is the MyD88/NF-κB axis (Fig. 4B; Table S2). The regulation of MyD88 by SPOP is context-dependent and occurs through two distinct molecular mechanisms. Firstly, upon TLR4 activation, SPOP translocates to the cytoplasm and is recruited to the receptor complex, where it binds to MyD88 and disrupts its self-association without triggering its degradation.^89^ This ubiquitination-independent mechanism rapidly inhibits the recruitment of downstream interleukin-1 receptor-associated kinases (IRAKs), providing an immediate brake on inflammatory signaling.^89^ Secondly, in a different context, such as during bacterial infection, SPOP acts as a typical E3 ubiquitin ligase adaptor, mediating the K48-linked polyubiquitination and proteasomal degradation of MyD88.^8^^,^^117^ This degradative pathway reduces the overall cellular level of MyD88, serving as a sustained mechanism to reset immune homeostasis.^8^^,^^117^ Thus, SPOP employs both non-degradative and degradative mechanisms to finely tune MyD88-dependent immune responses. In contrast, SPOP also leads to Newcastle disease virus replication in chickens by inhibiting MyD88/NF-κB-mediated production of pro-inflammatory factors.^7^ However, in most cases, SPOP suppresses viral replication through inflammation and immune responses. SPOP inhibits spring viremia of carp virus, HBV infection, and enterovirus 71 by mediating the ubiquitination and degradation of mitochondrial antiviral signaling protein (MAVS),^9^ hepatocyte nuclear factor 1alpha (HNF1α),^118^ and enterovirus 71 (EV71)-2Apro, respectively.^119^

SPOP-mediated immune and inflammatory responses also play a key role in hematopoietic stem cells,^120^ chronic pancreatitis,^121^ and diabetic nephropathy.^122^ SPOP regulates the inflammatory activation of hematopoietic stem cells by triggering ubiquitination and degradation of MyD88.^120^ Similarly, SPOP inhibits the activation of the NF-κB/IL-6 signaling pathway through ubiquitination degradation of FAS-associated death domain-containing protein (FADD), thereby inhibiting the activation of mouse pancreatic stellate cells.^121^ As for diabetic nephropathy, SPOP promotes NLRP3 degradation by enhancing the K48 linked-polyubiquitination of NLRP3, thereby inhibiting the NLRP3 inflammasome and contributing to the management of diabetic nephropathy.^122^

### The effects of SPOP on nervous system disease

SPOP also plays an integral role in nervous system-related diseases, which are mainly related to inflammation (Fig. 4C; Table S2). The interaction between GPR30 and SPOP promotes the ubiquitination and degradation of NLRP3 mediated by SPOP, thus inhibiting the activation of inflammasomes and causing neuronal pyroptosis in ischemic stroke.^49^ However, Chinese medicine *Zuojinwan* reverses SPOP-mediated ubiquitination of MyD88, which inhibits excess pro-inflammatory factors, thus inhibiting the damage of hippocampal neurons and improving the abnormal activation of microglia.^123^ In addition, SPOP is necessary for dipeptide repeat protein (DPR) to exert toxicity in *C. elegans* without affecting DPR expression.^124^ However, the elimination of BRD, the substrate protein of SPOP, leads to inhibition of DPR toxicity. Thus, the role of SPOP in nematodes is similar to that of SPOP in kidney cancer, promoting amyotrophic lateral sclerosis and frontotemporal dementia.^124^ In the mouse spinal cord, SPOP also promotes ubiquitination and degradation of Gli3, but not Gli2, thereby inhibiting the Hh signaling pathway and ventral spinal cord lateralization.^125^

### The effects of SPOP on development

At present, SPOP has been shown to act in developmental events, such as embryo implantation,^126^ bone development,^127^ senescence,^128^ and DNA replication in keratinocytes (Fig. 4D; Table S2).^129^ Liu et al have shown that SPOP regulates decidualization of mouse endometrial stromal cells and that hormones regulate the expression of SPOP, suggesting that ubiquitination may be involved in embryo implantation.^126^ As for bone development, SPOP directly targets Gli3 for ubiquitination and degradation, then Ihh target genes (Ptch1 and Pthlh) are up-regulated, indicating that Hh signaling is activated.^127^ Similarly, SPOP mediates the ubiquitination degradation of the desumoylase SUMO-specific protease 7 (SENP7), which promotes the sumoylation, the subcellular localization, and the related gene silencing of HP1α, thereby leading to cellular senescence.^128^ In terms of non-cancerous keratinocyte HaCaT cells, the depletion of SPOP reduces the expression of DNA replication licensing factors Cdc10-dependent transcript 1 protein (CDT1) and cell division cycle 6 (CDC6), which induces the expression of p21, resulting in cell cycle stagnation in the G1/S phase, indicating that SPOP is essential for DNA replication in HaCaT cells.^129^

### Others

SPOP plays an important role in maintaining cell homeostasis (Fig. 4E; Table S2). For example, the ubiquitination and degradation of nucleoporin protein 153 (Nup153) mediated by SPOP in the nuclear envelope and some nuclear foci inhibit the localization of the spindle assembly checkpoint protein mitotic arrest deficiency 1 (Mad1) in the nuclear envelope, thereby regulating cell cycle progression and cell division.^130^ Furthermore, the orphan nuclear receptor SHP interacts with the trans-activating domain of X-box binding protein 1 (XBP1) to inhibit the polyubiquitination and degradation of XBP1 by SPOP, thereby regulating the endoplasmic reticulum stress response and maintaining endoplasmic reticulum homeostasis.^75^

In the process of myocardial fibrosis, SPOP-mediated ubiquitination and degradation of receptor of activated protein C kinase 1 (RACK1) contribute to the activation of Smad3, which promotes cardiac fibroblast proliferation, cardiac fibrosis, and collagen production.^131^ Moreover, SPOP inhibits fetal hemoglobin expression in a manner that is independent of the transcription suppressors B-cell lymphoma/leukemia 11A (BCL11A) and LRF/ZBTB7A, but is dependent on Cullin3 activity.^132^

### SPOP may be a molecular bridge

Through the Cox proportional hazards analysis of SPOP in The Cancer Genome Atlas (TCGA) database, overall survival, disease-specific survival, disease-free interval, and platinum-free interval-related results showed that SPOP expression was a risk factor in approximately one-third of cancers (Fig. S1). The effect of SPOP as a risk factor is particularly significant in lung cancer, liver cancer, kidney cancer, mesothelioma, glioma, and head and neck squamous cell carcinoma (Fig. S1), suggesting that SPOP may act as an oncoprotein. To explore the possible underlying mechanism of SPOP as an oncoprotein, we further performed gene set enrichment analysis (GSEA) of SPOP in the TCGA database, and the results indicated that SPOP may be related to the activation of transcription (based on MYC TARGETS V1, MYC TARGETS V2, E2F TARGETS, etc.), immunity and inflammation (based on INFLAMMATORY RESPONSE, INTERFERON RESPONSE, etc.), and DNA replication and repair (based on G2/M CHECKPOINT, DNA REPAIR, etc.) (Fig. S2–S5).

The structure of SPOP and its speckled nuclear distribution^133^ provide a rationale for its function: the MATH domain recruits SPOP-interacting proteins, while the BTB and BACK domains facilitate interactions among these proteins, and the NLS domain ensures the occurrence of these events within the nucleus. Considering that SPOP accumulates in the nucleus in a speckle-type pattern^133^ providing some rationale for the generation of interactions between SPOP-enriched proteins, and that most SPOP proteins interact with oncoproteins,^11^ the role of SPOP as a molecular bridge is likely to exert its cancer-promoting effects. In 2022, Tawaratsumida et al have proposed that SPOP acts as a molecular bridge connecting Toll-like receptor (TLR) and C-terminal Src kinase (CSK).^134^ TLR recognizes pathogen and host-derived factors and controls immune responses through MyD88 and IRF family members, which coordinate key effector functions, including cytokine release, cell differentiation, and, in some cases, inflammatory pathology.^134^ However, IRF activity is controlled by LYN, whose phosphorylation of TLR-induced IRF leads to K48-linked polyubiquitination and proteasome-mediated degradation of IRF.^134^ The activity of LYN is controlled by CSK, and the activity of CSK is controlled by SPOP, which acts as a molecular bridge to recruit CSK into the TLR signaling complex and activate the catalytic activity of CSK.^134^ We hypothesize that SPOP might serve as a molecular bridge to facilitate the interaction between SPOP-interacting proteins.

Therefore, we propose that SPOP may act as a molecular bridge to facilitate the construction of multiple platforms, including transcription, immune/inflammation, and DNA replication/repair platforms (Fig. 5).

**Figure 5:**
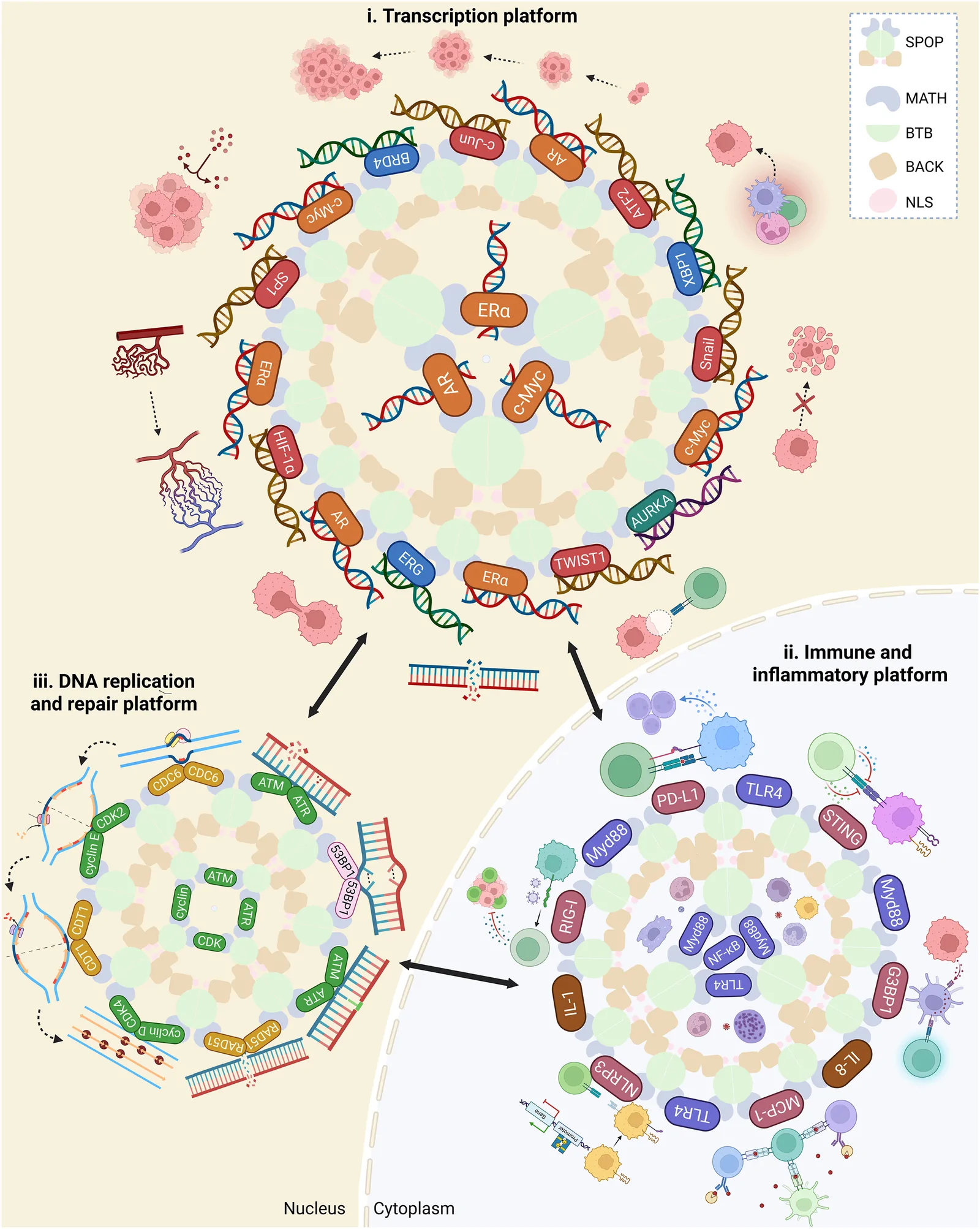
SPOP functions as a molecular bridge to assemble three distinct functional platforms. Our model proposes that SPOP, through its multi-domain structure capable of simultaneous protein interactions, serves as a central molecular scaffold to nucleate the formation of higher-order molecular platforms. These platforms spatially coordinate key cellular processes: i) The transcription platform, centered on the core transcription factors c-Myc, AR, and ERα, which are recruited by SPOP. This platform potentially integrates diverse oncogenic transcriptional signals and their complex cross-talk to fine-tune gene expression programs. ii) The immune/inflammation platform, organized around the canonical TLR4/MyD88/NF-κB signaling axis. Here, SPOP acts as a regulatory hub to bridge pathogen-sensing mechanisms with downstream inflammatory responses and immune checkpoint regulation. iii) The DNA replication/repair platform, coordinated by the key cell cycle regulators cyclin/CDK and the DNA damage sensors ATM/ATR. This platform positions SPOP to directly link cell cycle progression with the DNA damage response machinery.

### The transcription platform based on c-Myc, AR, and ERα

Classifying the proteins that interact with SPOP, transcription factors and transcription regulators account for the majority. Among the transcription-related proteins interacting with SPOP, c-Myc, AR, and ERα serve as core proteins on the constructed transcription platform.

### Dimer and heterodimer

Both AR and ERα activation require the formation of receptor dimers that mediate transcription of their downstream target genes.^135^^,^^136^ Generally, the stimulation of AR and ERα to form dimers is mediated by their own ligands.^135^^,^^136^ The molecular bridge constructed by SPOP, AR, or ERα captured by the MATH domain^137^^,^^138^ has the opportunity to form dimerization of AR or ERα through SPOP-BTB domain-mediated dimerization or SPOP-BACK-mediated oligomerization, thus promoting the construction of the cornerstone of the transcription platform. Interestingly, in ERα-positive breast cancer, AR and ERα can form AR–ERα heterodimers, thus suppressing breast cancer progression.^139^

As for c-Myc, it mediates transcription not by forming dimers, but by forming heterodimers with Max.^140^ c-Myc forms heterodimers with Max containing the same basic helix-loop-helix leucine zipper (BHLH-ZIP) structure through the C-terminal BHLH-ZIP structure, and then c-Myc-Max dimer binds to DNA to regulate transcription.^140^ In addition, it has recently been reported that c-Myc retains considerable biological activity without Max.^141^ However, the molecular nature of this Max-independent c-Myc activity may be multifaceted.^142^ For instance, Gallant et al discovered that Max was not required for c-Myc-activated RNA polymerase III-dependent transcription, suggesting that c-Myc may also support the transcription platform on its own.^141^

### Interaction among c-Myc, AR, and ERα

In addition to the dimer and heterodimer composition, c-Myc, AR, and ERα have complex crosstalk. Firstly, the regulatory mechanism of gene transcription between c-Myc and AR has been intricate; that is, its effects are tissue-specific and cell-specific, and can be triggered or inhibited.^143^ Moreover, the interplay between AR and c-Myc is complex and non-reciprocal. While AR predominantly suppresses c-Myc signaling, c-Myc exerts dual and opposing effects on AR: it inhibits AR's transcriptional activity by competing for shared co-factors, yet it can also promote AR gene expression and protein stability under certain contexts.^142^ Overexpression of c-Myc significantly reduces the transcriptional activity of AR, resulting in the suppression of transcription of downstream target genes of AR without altering the expression of *AR*.^144^ After c-Myc overexpression, there was an increase in AR-targeted genes that were suspended at the proximal end of the RNA polymerase II promoter, while AR binding enhancers were not accompanied by inactivation.^144^ Interestingly, c-Myc can bind to c-Myc binding sites on *AR* enhancers, promoting transcription of *AR* and enhancing the stability of AR proteins.^145^ In addition, c-Myc overexpression led to a marked increase in AR binding sites, and c-Myc is abundant in a subset of AR binding sites.^146^ On the contrary, the effect of AR on c-Myc is relatively clear, which is manifested as inhibition. When the AR is heavily activated by androgens, transcriptional coactivators in the nucleus are depleted of activated AR, which contributes to a decline in transcriptional coactivators (such as BRD4) at the chromatin binding site of c-Myc.^146^ In addition, activated AR can directly inhibit the transcriptional activation of c-Myc, thereby suppressing the expression of cell proliferation-related genes (c-Myc target genes).^146^

Second, the link between c-Myc and ERα was bidirectional. Estrogen enhances the interaction between c-Myc and ERα, which promotes the formation of the c-Myc–ERα–coactivator complex to the estrogen response promoter, leading to chromatin remodeling and increased transcription.^147^ In addition, the distal enhancer element in the 67 kb region upstream of the transcription start site of *c-Myc* is an ERα-mediated *c-Myc* transcription enhancer.^148^ Estrogen up-regulates *c-Myc* gene expression through the cooperation between ERα and regulatory factors such as activator protein 1 (AP-1) on this distal enhancer.^148^

Third, the interaction between AR and ERα was the clearest among the three. AR is closely related to ERα, and it has a signaling crosstalk effect in breast cancer.^149^ The N-terminus of AR can bind to the ligand-binding domain of ERα,^150^ and AR and ERα have common coactivating proteins.^149^ Ligand activation or continuous overexpression of AR can reduce the activity of ERα and inhibit the growth of breast cancer cells,^149^^,^^150^ and this suppressive effect requires the existence of the DNA-binding domain of AR. Notably, several studies have detected a large number of activated AR clustered at the estrogen response elements, which indicates that AR may competitively bind to the estrogen response elements with ERα, thereby exerting an inhibitory effect.^149^, ^150^, ^151^

In summary, we believe that a transcription platform based on c-Myc, AR, and ERα may be constructed on the molecular bridge supported by SPOP. Moreover, the interaction between c-Myc, AR, and ER and their crosstalk may further promote the stability and function of the transcription platform.

### Interactions between c-Myc/AR/ERα and other transcriptional regulators

Based on these three core proteins, there are ten other important transcriptional regulators, including BRD4, XBP1, AURKA, ERG, Twist-related protein 1 (TWIST1), Snail, HIF-1α, c-Jun, specificity protein 1 (SP1), and activating transcription factor 2 (ATF2). These 10 proteins are not only closely related to SPOP but also to c-Myc, AR, and ERα; thus, they may cooperate with the molecular bridge and the three core proteins to further refine the transcription platform. Based on the relationships between these proteins and c-Myc, AR, and ERα, they can be divided into three categories: upstream regulators, downstream regulators, and cross-regulators of core proteins.

There are three upstream regulators of the core proteins: BRD4, XBP1, and ERG. Among them, BRD4 and XBP1 showed transcriptional promoting effects on c-Myc, AR, and ERα.^152^, ^153^, ^154^, ^155^, ^156^, ^157^ In PCa, ERG overexpression activates c-Myc and eliminates prostate epithelial differentiation.^158^ ERG promotes AR signaling by maintaining a co–regulator complex at the AR binding site,^159^ but the transcriptional activity of ERG is inhibited by ERα.^160^ In contrast, ERG is also able to inhibit ERα-mediated transcription of target genes.^160^ The antagonism between ERG and ERα is not dependent on DNA binding inhibition or protein–protein interactions.^160^ Inhibition of ERα-mediated transcription of target genes requires the C- and N-terminal trans-activating domains of ERG, and the C-terminal AF-2 domain of ERα is required for the inhibition of ERG.^160^

AURKA, the only downstream regulator of core proteins, is subject to the up-regulation of c-Myc, AR, and ERα. Both c-Myc and AR up-regulate AURKA transcription by binding to specific binding regions in the *AURKA* promoter.^161^^,^^162^ However, ERα up-regulates AURKA through ERα–GATA binding protein 3 (GATA-3)–AURKA signaling.^163^

Among the remaining cross-regulators of core proteins, c-Myc generally acts as the upstream protein of the cross-regulators, AR as the downstream promoting protein, and ERα as the downstream inhibiting protein. For example, c-Myc binds to and activates *Snail* and *TWIST1* promoters, thereby promoting Snail and TWIST1 expression and thus mediating metastasis.^164^, ^165^, ^166^ In contrast, c-Myc inhibits Ras-mediated differentiation of pheochromocytoma cells by blocking the up-regulation of c-Jun through its DNA-binding and transcription-activating regions.^167^ In addition, c-Myc is able to regulate the activity of ATF2 and HIF-1α through post-translational modifications, such as phosphorylation.^168^^,^^169^ c-Myc does not appear to have a direct effect on SP1, but it cooperates with the transcriptional regulation of their target genes.^170^ For AR, most cross-regulators, including Snail,^63^ c-Jun,^171^ SP1,^172^ Twist1,^173^ Snail,^174^ and HIF-1α,^175^ promote AR activation by binding to specific binding regions on *AR* promoters, leading to AR dimerization and DNA binding. Similarly, Snail and c-Jun inhibit *ESR1* transcription by binding to their promoters.^176^^,^^177^ In addition, ATF2 reduces the levels of ERα and ERα regulatory genes at the RNA and protein levels.^178^ However, TWIST1 does not directly inhibit ERα, but down-regulates the expression of ERα in breast cancer through miR-22.^179^

### The immune and inflammatory platform based on TLR4/MyD88/NF-κB

Among the remaining interacting proteins of SPOP, immune- and inflammation-related proteins are the main components that form a platform of immunity and inflammation. In this platform, the TLR4/MyD88/NF-κB signaling pathway is the skeleton, accompanied by interweaving of the cGAS/STING signaling pathway and crosstalk between downstream proteins.

### TLR4/MyD88/NF/κB signaling pathway

As a member of the pattern recognition receptors (PRR), TLR recognizes specific patterns of microbial components.^180^ Macrophages and dendritic cells use PRR to respond to bacteria and viruses, thus playing an important role in innate immunity.^181^ Among these PRR, TLR is a central protein in immune responses by recognizing pathogen-associated molecular patterns (PAMPs) from bacteria and viruses, as well as from dead or damaged cells.^181^ TLR is specific for its ligand recognition, and TLR4 mainly recognizes lipopolysaccharide.^182^ When TLR4 is activated, there are two signaling pathways: the MyD88-dependent signaling pathway and the MyD88-independent signaling pathway.^182^ The MyD88-dependent signaling pathway is induced by the binding of the activated TIR domain to MyD88, which promotes the recruitment of IRAK1, IRAK4, and TRAF6 to the TLR4–MyD88 complex and results in the phosphorylation of IRAK1 and TRAF6.^182^ After this, the signal is propagated from the phosphorylated adaptor complex to the downstream MAP kinase-AP1 and IKK complex NF-κB, inducing the expression of related genes, thereby resulting in immune and inflammatory responses.^182^^,^^183^

SPOP, as the molecular bridge of the TLR4/MyD88/NF-κB signaling pathway, runs through the entire signaling pathway. It is closely related not only to TLR4, MyD88, and NF-κB, but also to downstream proteins, such as PD-L1, NLRP3, RIG-I, and monocyte chemoattractant protein-1 (MCP-1).^89^^,^^120^^,^^123^ When TLR4 is activated, SPOP is recruited to MyD88 and promotes the transfer of SPOP from the nucleus to the cytoplasm, thereby disrupting the self-association of MyD88 without affecting its ubiquitination and degradation,^89^ inhibiting the recruitment of downstream kinases, thereby limiting the inflammatory response.^89^ In addition, SPOP can not only down-regulate MyD88 through poly-ubiquitination and degradation,^120^ but also block the assembly of MyD88 through non-degrading ubiquitination.^184^ Subsequently, the activity of NF-κB is inhibited, which regulates the expression of downstream target genes and controls immune and inflammatory homeostasis.^123^ As for NF-κB, SPOP generally inhibits its activity. On the one hand, SPOP inhibits the NF-κB signaling pathway by down-regulating the expression of p-PI3K, p-AKT, and NF-κB^185^; on the other hand, it suppresses the activation of NF-κB by promoting ubiquitination and degradation of FADD.^121^^,^^186^

### The crosstalk with the cGAS/STING signaling pathway

In this immune and inflammatory platform, there is another signaling pathway related to innate immunity, that is, the cGAS/STING signaling pathway, which not only contains many interacting proteins of SPOP (such as STING and G3BP1), but is also closely related to the TLR4/MyD88/NF-κB signaling pathway.

When cGAS dimerizes with abnormal double-stranded DNA, it catalyzes ATP and GTP to form cyclic GMP-AMP (cGAMP), which acts as a second messenger to transmit signals to STING, resulting in conformational changes in STING and transporting to the Golgi apparatus.^187^ Subsequently, with the help of palmitoacyltransferase, STING recruits and phosphorylates TANK-binding kinase 1 (TBK1), which leads to phosphorylation of STING, recruiting and phosphorylating IRF3.^188^ Phosphorylated IRF3 enters the nucleus to induce expression of type I interferon and interferon-stimulated genes.^187^, ^188^, ^189^ At the same time, the cGAS/STING pathway can activate the NF-κB signaling pathway, and promote the production of pro-inflammatory cytokines such as interferon, interleukin, and tumor necrosis factor.^189^ In addition, the cGAS/STING signaling pathway in monocytes can not only cooperate with MyD88 to promote the production of pro-inflammatory factors during bacterial infection,^190^ but also recruit MyD88 to induce the p38-dependent signaling pathway to produce IL-6, thus inducing the occurrence of inflammation.^191^ When extracellular cold-induced RNA-binding proteins are released from the cells, the TLR4/MyD88 signaling pathway activates the cGAS/STING signaling pathway, exacerbating inflammation.^192^ Thus, the cGAS/STING signaling pathway and the TLR4/MyD88/NF-κB signaling pathway can promote each other, synergistically leading to inflammation.

### The crosstalk between downstream proteins

There are intricate interactions between the four downstream proteins of the TLR4/MyD88/NF-κB signaling pathway, the most important of which are PD-L1 and NLRP3.

First, the activation of NLRP3 leads to high levels of PD-L1, resulting in oxaliplatin resistance and remodeling of the immunosuppressive microenvironment.^193^ Similarly, NLRP3-mediated inflammasome activation in diffuse large B-cell lymphoma promotes PD-L1 expression and reduces the proportion of cytotoxic T cells, thus promoting immunosuppression.^194^ In contrast, PD-L1 negatively regulates NLRP3, thereby inhibiting NLRP3-mediated pyroptotic cell death, which leads to cisplatin resistance in gastric cancer cells.^195^

Second, the effects of RIG-I and MCP-1 on PD-L1 expression were reversed. RIG-I competitively binds to PD-L1 with SPOP, resulting in decreased polyubiquitination and proteasome degradation of PD-L1, thereby maintaining the stability of PD-L1 in colorectal cancer and driving immune escape.^68^ In mesenchymal stem cells, MCP-1 inhibits the TLR4/MyD88/NF-κB/PD-L1 signaling pathway to avoid T cell apoptosis, thereby enhancing T cell activation in lymph nodes.^196^

Finally, RIG-I and MCP-1 participate in the regulation of NLRP3 signaling upstream and downstream of NLRP3, respectively. Influenza A viruses can activate the RIG-I/TLR3/NLRP3 signaling pathway, initiating inflammasome activation.^197^ However, activation of the NLRP3 inflammasome significantly increases the production of MCP-1 in vascular endothelial cells.^198^

### The DNA replication and repair platform based on cyclin/CDK and ATM/ATR

As a molecular bridge, SPOP also gathers proteins related to DNA replication and repair, thereby building the DNA replication and repair platform and regulating DNA homeostasis. This platform is composed of two parts: cyclin/CDK and ATM/ataxia telangiectasia and Rad3-related protein (ATR).

### cyclin/CDK and ATM/ATR

DNA replication is mediated by the cell cycle, consisting of G1, S, G2, and M phases.^199^ To ensure that the genetic material is correctly copied and accurately transmitted to the next generation, long-term evolution has made the cell have a strict set of periodic checkpoints.^199^ The G1, S, and G2 checkpoints are closely associated with DNA damage.^199^ Among these, the checkpoints of the G1 phase are closely related to SPOP. Both ATM and ATR at the G1 checkpoint can interact with SPOP, and cyclin and CDK in the G1 phase can bind to SPOP. Thus, the DNA replication and repair platform promoted by SPOP mainly occurs in the G1 phase.

Stimulated by growth factors, cells emerge from dormancy and enter the G1 phase.^200^ With the initiation of the cell cycle, cyclin D is rapidly synthesized and interacts with CDK4/6, which promotes the phosphorylation of retinoblastoma tumor suppressor gene (RB), thereby inducing the dissociation of RB from E2F.^200^ The E2F enters the nucleus and promotes the transcription of cyclin E and the genes required to enter the S phase.^200^ In addition, the activated cyclin D–CDK4/6 complex interacts with p21 Cip and p27 Kip, eliminating the inhibitory effect of p21 Cip and p27 Kip on cyclin E–CDK2.^200^ The aggregation of cyclin E–CDK2 complex further catalyzes RB phosphorylation, triggering DNA replication-related gene transcription and G1–S phase transition.^200^ However, DNA damage activates ATM/ATR-mediated CHK phosphorylation, which promotes ubiquitination and degradation of phosphatase cell division cycle 25A (CDC25A), resulting in CDK2-cyclin E and CDK2–Cyclin A inactivation, contributing to cell cycle arrest.^199^

SPOP, as a molecular bridge, is the basis of the DNA replication and repair platform and strongly interacts with ATM/ATR and cyclin/CDK. ATM/ATR and cyclin/CDK usually cooperate with SPOP to promote DNA replication and repair. When DNA is damaged, SPOP is recruited to the DNA double-strand breaks and forms nuclear lesions.^201^ With the help of ATM, S119 in SPOP is phosphorylated, resulting in conformational changes in SPOP, which enhance the non-degrading ubiquitination of 53BP1 ^41^ and HIPK2,^42^ as well as weaken the ubiquitination and degradation of minichromosome maintenance complex component 3 (MCM3),^88^ thereby regulating cell cycle progression and DNA damage repair. Similarly, down-regulation of SPOP is associated with reduced transcription levels of DNA repair and replicator factors, including ATR, breast cancer type 1 susceptibility protein (BRCA1), checkpoint kinase 1 (CHK1), and RAD51.^202^ As for the cyclin/CDK complex, Cyclin D–CDK4-mediated SPOP phosphorylation disrupts its binding to FZR1 through recruitment of 14-3-3γ, thereby inhibiting FZR1-mediated SPOP degradation.^36^ In addition, CDK2 competitively binds to the MATH domain of SPOP with Cyclin E, thus inhibiting SPOP-mediated polyubiquitination and degradation of Cyclin E1, promoting the accumulation of Cyclin E1.^84^ Therefore, SPOP serves as a molecular bridge to construct the DNA replication and repair platform based on ATM/ATR and cyclin/CDK.

### The crosstalk between cyclin/CDK and ATM/ATR

First, it is well documented that ATM/ATR has an inhibitory effect on cyclins/CDKs. ATM/ATR plays a role in the cell cycle by regulating the initiation and progression of DNA synthesis in preparation for stopping replication in the face of DNA damage.^203^ Random endogenous replication stress induces CDK2 activity fluctuations triggered by ATM/ATR, thereby dynamically adjusting the global DNA synthesis rate.^204^ When DNA is damaged, such as exposure to ionizing radiation, ATM/ATR is activated to inhibit CDK2, delaying DNA replication to prevent the formation of drug-resistant DNA synthesis.^205^^,^^206^ Similarly, inhibition of ATM/ATR aggravates Cyclin E-induced mitosis.^207^ Therefore, tumor-specific alterations, such as ATM/ATR loss and Cyclin E1 amplification, are more sensitive to ATM/ATR inhibitors, including elimusertib and M1774.^208^ In addition, the interaction between ATM/ATR and Cyclin D achieves genomic integrity by rapidly halting the cell cycle, and disruption of this interaction may increase susceptibility to cancer.^209^ When ATM/ATR is inhibited, Cyclin D–CDK4 drives genomic instability.^209^^,^^210^ Conversely, ATM/ATR-mediated phosphorylation promotes the degradation of Cyclin D–CDK4, thereby ensuring the cell cycle.^211^ In neurons, ATM is also able to inhibit the expression of Cyclin D, thereby inhibiting the entry of abnormal cell cycles and thus maintaining differentiated neurons.^211^

Second, cyclin/CDK has a promoting effect on ATM/ATR, whether by cyclin/CDK inhibition or cyclin/CDK activation. Inhibition of CDK2 prompts the MCM complex to bind to DNA and trigger re-replication, inducing DNA double-strand breaks and the replication protein A (RPA)–ssDNA intermediates, which activate ATM/ATR, thereby selectively deleting cells with re-replication.^203^ Moreover, the inhibition of CDK2 induces the p53–p21 response through ATM/ATR-dependent phosphorylation of p53, inducing DNA re-replication, and maintaining genomic stability.^212^ Uniformly, the activation of Cyclin E1–CDK2 triggers replication stress and activates ATR and chromosome 1 (CHR1)-dependent cell cycle arrest.^213^^,^^214^ In addition, sustained CDK4/6 activation drives into the S phase, which causes replication stress, DNA damage, and ATM/ATR activation.^215^ CDK4/6-mediated phosphorylation of Reprimo (RPRM) promotes nuclear translocation of RPRM, thereby facilitating its interaction with ATM, leading to nuclear output.^216^

Thirdly, Cyclin D–CDK4 and Cyclin E–CDK2 promote each other and jointly regulate DNA replication. The promotion of Cyclin D–CDK4 by Cyclin E–CDK2 is reflected in two aspects. On the one hand, EGF-mediated activation of CDK4 induces RB inactivation and E2F activation, which drives Cyclin E–CDK2 and coordinates the G1/S transition.^217^, ^218^, ^219^ On the other hand, Cyclin D promotes CDK2 phosphorylation at T160, triggering the formation of Cyclin D–CDK2 complex.^220^ P21, as a scaffold protein, further promotes the formation of Cyclin D–CDK2–PCNA trimers, mediating the phosphorylation of various substrates.^221^ In addition, the formation of this trimer inhibits DNA replication and CDK2 activity.^222^ However, there are few studies on the promotion effect of Cyclin E–CDK2 on Cyclin D–CDK4. CDK2 promotes centrosome over-replication and proliferation of hepatocytes with high expression of Cyclin D,^223^ and down-regulation of CDK2 inhibits CDK4-mediated tumorigenesis.^224^

### ATM/ATR-mediated DNA replication and repair

In addition to its association with cyclin/CDK, ATM/ATR regulates DNA replication and repair through several other proteins, including 53BP1 and RAD51 (DNA repair) and CDC6 and CDT1 (DNA replication). These proteins are not only SPOP-interacting proteins, but also ATM/ATR and cyclin/CDK-interacting proteins, which together with ATM/ATR and cyclin/CDK make up this DNA replication and repair platform.

In general, ATM/ATR promotes DNA repair through 53BP1 and RAD51. In turn, 53BP1 promotes ATM/ATR-mediated DNA repair. When exposed to ionizing radiation or hypoxia, DNA breaks, which activate ATM/ATR, thereby triggering the phosphorylation of 53BP1 to ensure genomic stability.^225^ In turn, 53BP1 is rapidly recruited to the DNA double-strand break sites, where it acts as a DNA damage checkpoint to induce cell cycle arrest and activate ATM.^226^ In addition, 53BP1 amplifies the interaction between the MRN and ATM by interacting directly with the MRN complex, promoting the repair of DNA double-strand breaks.^227^ As for ATR, 53BP1 protects the replication fork through ATR–CHK1–p53 signaling, which promotes DNA damage repair.^228^ Under the induction of ionizing radiation, ATM/ATR can also induce the formation of RAD51 nuclear filaments, and accompanied by the isolation of RAD51 from the nuclear matrix, homologous recombination is triggered.^229^^,^^230^ ATR can partially compensate for the early homologous recombination after ATM inhibition.^230^ Moreover, ATR-mediated de-ubiquitination of RAD51 promotes the interaction of RAD51 with BRCA2, which promotes the recruitment of 53BP1 to DNA double-strand break sites, thus promoting homologous recombination.^231^

ATM/ATR has an inhibitory effect on DNA replication-related proteins (CDC6 and CDT1). CDC6, on the one hand, promotes ATM/ATR-mediated apoptosis and, on the other hand, activates the ATR–CHK1 pathway to promote cisplatin resistance.^232^^,^^233^ In addition, overexpression of CDT1 can induce re-replication of cancer cells, thereby activating the ATM/ATR-dependent checkpoint pathway.^234^ In contrast, ATM/ATR promotes the degradation of CDC6 and CDT1, thereby preventing DNA re-replication to maintain genomic stability.^235^, ^236^, ^237^

### cyclin/CDK-mediated DNA replication and repair

The correlation between cyclin/CDK and DNA repair-related proteins is mainly reflected in the promotion effect of Cyclin D on RAD51. Under radiation, Cyclin D promotes the recruitment of RAD51 by BRCA2-containing DNA repair foci, thereby promoting the expression of RAD51 and the formation of nuclear lesions to induce homologous repair, thus ensuring the integrity of DNA.^238^, ^239^, ^240^ However, CDK2-mediated phosphorylation of BRCA2 limits BRCA2's contact with RAD51, resulting in an impaired replication fork and cleavage of newly synthesized DNA.^241^

The relationship between cyclins/CDKs and DNA replication proteins is mainly reflected in the interaction between CDC6 and cyclins/CDKs. Most importantly, CDC6 mainly promotes cyclin/CDK. Ectopic overexpression of CDC6 promotes Cyclin D expression, CDK4 activation, and G1 progression.^242^ Uniformly, knockout of CDC6 reduces the levels of Cyclin D, thereby inhibiting proliferation.^243^ Additionally, CDC6 interacts with CDK2 to promote the activity of CDK2, thus playing an important role in Cyclin E–CDK2 activation, DNA synthesis initiation, and G1–S phase transition.^244^ CDC6 also activates CDK2, but this only occurs in the CDK2 protein that binds to p27^245^ or in the CDK2 protein associated with p21.^246^ In general, cyclin/CDK promotes CDC6. On the one hand, CDK2-mediated phosphorylation of CDC6 promotes the entry of CDC6 into the cytoplasm, thereby preventing the re-replication of S and G1 phases,^247^ and on the other hand, it protects CDC6 from CDH1-mediated degradation, thereby promoting replication.^248^ Similarly, CDK4 up-regulates CDC6 via the CDK4–RB–CDC6 signaling pathway, thus promoting DNA replication and inhibiting apoptosis.^249^

## Discussion

In conclusion, we elucidated four different domains of SPOP, which are the structural basis of the molecular bridge formed by SPOP. Next, the promotive and inhibitory regulation of SPOP is also described, mainly regarding the changes in the expression and activity of SPOP. We then show the roles of SPOP in cancer inhibition, cancer promotion, nervous system diseases, immunity, inflammation, and development. Finally, we focus on the three platforms that may depend on SPOP as a molecular bridge, as well as the crosstalk between the three platforms.

Our proposed SPOP-constructed molecular bridge is a preliminary hypothesis. The transcription platform, immune/inflammation platform, and DNA replication/repair platform built by SPOP only highlight the key scaffold proteins. In the transcription platform, there are ten other potential transcriptional regulators, including BRD4, XBP1, AURKA, ERG, TWIST1, Snail, HIF-1α, c-Jun, SP1, and ATF2.^152^, ^153^, ^154^, ^155^, ^156^, ^157^^,^^164^, ^165^, ^166^ These 10 proteins are not only closely related to SPOP but also to c-Myc, AR, and ERα; thus, they may cooperate with the molecular bridge and the three core proteins to further refine the transcription platform. In the immune/inflammatory platform, based on the TLR4/MyD88/NF-κB signaling pathway, there are four main downstream proteins, namely PD-L1, NLRP3, MCP-1, and RIG-I, which interact with SPOP proteins and jointly regulate the immune and inflammatory environment.^250^, ^251^, ^252^, ^253^, ^254^ In the DNA replication/repair platform, ATM/ATR regulates DNA replication and repair through several other proteins, including 53BP1 and RAD51 (DNA repair) and CDC6 and CDT1 (DNA replication).^225^^,^^228^^,^^232^^,^^233^ These proteins are not only SPOP-interacting proteins, but also ATM/ATR and cyclin/CDK-interacting proteins, which together with ATM/ATR and cyclin/CDK make up this DNA replication and repair platform.^225^^,^^228^^,^^232^^,^^233^

Further GSEA analysis of the genes associated with the three platforms built by SPOP revealed that the activation or inhibition of the three platforms was cancer-specific (Fig. S6–S9). In most cancers, these three platforms are not activated or inhibited simultaneously but are partially activated or partially suppressed (Fig. S6–S9). Intricately, related gene sets inside the transcription platform also present the opposite situation. For example, in breast invasive carcinoma, the ERα-associated gene sets in the transcription platform are activated, whereas the c-Myc-associated gene sets are inhibited, which is also observed in testicular germ cell tumors (Fig. S6–S9). In general, the transcription platform showed opposite trends in gene sets associated with DNA replication and repair. For instance, the gene sets associated with the transcription platform were inhibited, whereas the gene sets associated with the DNA replication and repair platform were activated in cholangiocarcinoma (Fig. S6–S9). In addition, the results of GSEA analysis showed that transcription platform-related gene sets were mostly suppressed, while DNA replication and repair-related gene sets were activated, which may be attributed to the relationship between the molecular bridge SPOP and the proteins of the platform (Fig. S6–S9). For the transcription platform, not only can the core proteins (c-Myc, AR, and ERα) be degraded by SPOP, but most of the other proteins are negatively regulated by SPOP, so the transcription platform may be inhibited in most cancers.^11^ In contrast, ATM and ATR in the DNA replication and repair platform are upstream positive regulatory proteins of SPOP, which may further promote the construction of the DNA replication and repair platform, thus enabling it to be activated in cancers.^11^ Moreover, the GSEA results point that three platforms are simultaneously suppressed in more than a dozen cancers, including pancreatic adenocarcinoma and sarcoma, while simultaneous inhibition was only seen in low-grade glioma and pan-kidney cohorts (Fig. S6–S9), which may be due to the functional specificity of SPOP in different cancers and its ability to mediate substrate ubiquitination and degradation. After analyzing the correlation between key proteins in the three transcription platforms and SPOP, we found that SPOP was positively correlated with most of these proteins, which vary in different cancers (Fig. S10–S12). For example, the expression of SPOP was positively correlated with the expression of the core proteins of the transcription platform (c-Myc, AR, and ERα) in RCC, but not in HCC, suggesting that the activity of the platforms is cancer-specific (Fig. S10–S12).

To date, research on SPOP has been confined to preclinical studies, and no phase 1 clinical trials have been conducted. However, there are abundant studies on the sensitivity of SPOP to chemotherapy as well as the resistance of cancer-derived SPOP mutations to chemotherapy. For example, SPOP mitigated the development of docetaxel resistance in PCa by mediating the ubiquitination and degradation of ELK3.^101^ However, PCa-derived SPOP mutations F125V and F133L lost the ability to degrade caprin1, enhancing the assembly of stress granules, resulting in PCa resistance to docetaxel-induced cellular stress.^255^ However, the effects of SPOP on radiotherapy are different from those of chemotherapy. Given that SPOP promotes DNA damage repair by up-regulating the expression of DNA damage response factors, SPOP knockout and PCa-associated SPOP mutations down-regulate RAD51 and CHK1 mRNA to impair DNA damage repair, thus improving PCa sensitivity to radiotherapy.^256^ Although surgery, radiation therapy, endocrine therapy, and chemotherapy are all important components of PCa treatment, radical surgical resection and radical radiation therapy are relatively more effective.^257^ Compared with surgery, radiation therapy is recommended for PCa because of its low risk and high acceptability.^258^ Therefore, identifying or developing several potential SPOP-targeting inhibitors may have more clinical and practical value for the radical treatment of PCa. Surgery is the cornerstone of treatment for EC, with radiotherapy, chemotherapy, and endocrine therapy serving as postoperative adjuvant therapies. Therefore, whether SPOP inhibitors or agonists are used in EC may depend on factors such as the patient's physical state, side effects of the drugs, and treatment strategy used.

In addition to these three molecular platforms, GSEA analysis also showed that SPOP may be involved in the activation of gene sets related to oxidative phosphorylation and protein secretion, indicating that SPOP may also participate in the construction of other platforms, such as oxidative phosphorylation- or endocrine-related platforms (Fig. S2–S5). Unfortunately, most known SPOP-interacting proteins are not strongly linked to these processes, and few studies have focused on such connections. Harper et al reported that PTEN-induced putative kinase 1 (PINK1)/Parkin-mediated ubiquitin chains promoted autophagy in damaged mitochondria, thereby regulating oxidative phosphorylation, suggesting that ubiquitination may be involved in oxidative phosphorylation.^259^ However, whether SPOP-mediated ubiquitination participates in oxidative phosphorylation or the relationship between SPOP and oxidative phosphorylation-related proteins remains to be further explored. Furthermore, the molecular bridge model proposed here, while compelling, requires direct experimental validation. Future studies employing techniques such as proximity-dependent biotinylation (*e.g.*, BioID), advanced microscopy to visualize co-condensation, and the development of dimerization/oligomerization-deficient SPOP mutants will be crucial to test the physical existence of these proposed platforms and their functional impact in different cancer contexts.

## CRediT authorship contribution statement

**Yidong Ge:** Writing – original draft, Investigation. **Lili Kong:** Validation, Investigation. **Yuxuan Li:** Validation, Investigation. **Xiaofeng Jin:** Writing – review & editing, Funding acquisition, Conceptualization.

## Funding

This work was supported by the National Natural Science Foundation of Zhejiang, China (No. LY24C050001), the 10.13039/501100001809National Natural Science Foundation of China (No. 32270821, 32570835), Ningbo University High-Level Sci-Tech Project Incubation Program (Category III, GJPY2025009), the Natural Science Foundation of Ningbo, Zhejiang, China (No. 2024J037), and the K.C. Wong Magna Fund of Ningbo University (Zhejiang, China).

## Conflict of interests

The authors declared no competing interests.
