# Supplementary material for: Not only as a Cullin3 ligase adaptor, SPOP may also act as a molecular bridge
Source: Genes Dis. 2026 Apr 14;14(2):102189. doi: 10.1016/j.gendis.2026.102189 (PMC13659333; doi:10.1016/j.gendis.2026.102189)
Supplement: Supplementary file 1 [file mmc1.docx]

**Supplement Figure 1** The Cox proportional hazards analysis of SPOP based on TCGA database. The OS, DSS, DFI and PFI-related results showed that SPOP expression is a risk factor in approximately one-third of cancers.

**Supplement Figure 2** The GSEA analysis of SPOP based on TCGA database. The physiological processes of gene set enrichment associated with different expression of SPOP is distributed in Acute Myeloid Leukemia, Adrenocortical carcinoma, Breast invasive carcinoma, Cervical and endocervical cancers, Esophageal carcinoma, Glioblastoma multiforme, Liver hepatocellular carcinoma and Lung adenocarcinoma.

**Supplement Figure 3** The GSEA analysis of SPOP based on TCGA database. The physiological processes of gene set enrichment associated with different expression of SPOP is distributed in Bladder urothelial carcinoma, Brain Lower Grade Glioma, Cholangiocarcinoma, Colorectal adenocarcinoma, Glioma, Head and Neck squamous cell carcinoma, Lung squamous cell carcinoma and Lymphoid Neoplasm Diffuse Large B-cell Lymphoma.

**Supplement Figure 4** The GSEA analysis of SPOP based on TCGA database. The physiological processes of gene set enrichment associated with different expression of SPOP is distributed in Mesothelioma, Ovarian serous cystadenocarcinoma, Pheochromocytoma and Paraganglioma, Prostate adenocarcinoma, Stomach adenocarcinoma, Stomach and Esophageal carcinoma, Thyroid carcinoma and Uterine Carcinosarcoma.

**Supplement Figure 5** The GSEA analysis of SPOP based on TCGA database. The physiological processes of gene set enrichment associated with different expression of SPOP is distributed in Pancreatic adenocarcinoma, Pan-kidney cohort (KICH+KIRC+KIRP), Sarcoma, Skin Cutaneous Melanoma, Testicular Germ Cell Tumors, Thymoma, Uterine Corpus Endometrial Carcinoma and Uveal Melanoma.

**Supplement Figure 6** The GSEA analysis of three platforms-related physiological processes. The physiological processes of gene set enrichment associated with three platforms based on SPOP is distributed in Acute Myeloid Leukemia, Adrenocortical carcinoma, Breast invasive carcinoma, Cervical and endocervical cancers, Esophageal carcinoma, Glioblastoma multiforme, Liver hepatocellular carcinoma and Lung adenocarcinoma.

**Supplement Figure 7** The GSEA analysis of three platforms-related physiological processes. The physiological processes of gene set enrichment associated with three platforms based on SPOP is distributed in Bladder urothelial carcinoma, Brain Lower Grade Glioma, Cholangiocarcinoma, Colorectal adenocarcinoma, Glioma, Head and Neck squamous cell carcinoma, Lung squamous cell carcinoma and Lymphoid Neoplasm Diffuse Large B-cell Lymphoma.

**Supplement Figure 8** The GSEA analysis of three platforms-related physiological processes. The physiological processes of gene set enrichment associated with three platforms based on SPOP is distributed in Mesothelioma, Ovarian serous cystadenocarcinoma, Pheochromocytoma and Paraganglioma, Prostate adenocarcinoma, Stomach adenocarcinoma, Stomach and Esophageal carcinoma, Thyroid carcinoma and Uterine Carcinosarcoma.

**Supplement Figure 9** The GSEA analysis of three platforms-related physiological processes. The physiological processes of gene set enrichment associated with three platforms based on SPOP is distributed in Pancreatic adenocarcinoma, Pan-kidney cohort (KICH+KIRC+KIRP), Sarcoma, Skin Cutaneous Melanoma, Testicular Germ Cell Tumors, Thymoma, Uterine Corpus Endometrial Carcinoma and Uveal Melanoma.

**Supplement Figure 10** The correlation expression between SPOP and proteins involved in transcriptional platforms. Based on TCGA database, the expression of SPOP is positive related with proteins involved in transcriptional platforms in most cancers.

**Supplement Figure 11** The correlation expression between SPOP and proteins involved in immune and inflammatory platforms. Based on TCGA database, the expression of SPOP is positive related with proteins involved in immune and inflammatory platforms in most cancers.

**Supplement Figure 12** The correlation expression between SPOP and proteins involved in DNA replication and repair platforms. Based on TCGA database, the expression of SPOP is positive related with proteins involved in DNA replication and repair platforms in most cancers.

Supplement Table 1. The upstream regulator of SPOP.

| Upstream | Binding site | Modification on SPOP | Effects on SPOP | Effects on diseases | Reference |
| --- | --- | --- | --- | --- | --- |
| C/EBPα | -1144 to -1135, -815 to -806 and +55 to +64 on *SPOP* promoter | NA | promote SPOP transcription | Inhibit NSCLC | ^1^ |
| RXRA | -168 to -162 on *SPOP* promoter | NA | promote SPOP transcription | Inhibit CRC | ^2^ |
| ERG | *SPOP* promoter | NA | promote SPOP transcription | Inhibit PCa | ^3^ |
| HIF-1/2α | hypoxia-responsivecis-regulatory element | NA | promote SPOP transcription | Promote RCC and inhibit mobility of trophoblast cells | ^4,5^ |
| SMAD3 | SMAD-binding elements on *SPOP* promoter | NA | inhibit SPOP transcription | Promote PCa | ^6^ |
| NA | -160 to -27 on *SPOP* promoter | methylation | inhibit SPOP transcription | Promote NSCLC | ^1^ |
| LncRNA ADAMTS9-AS2 | NA | NA | Upregulate SPOP mRNA | Inhibit GC | ^7^ |
| circSLCO1B3 | NA | NA | Inhibit SPOP-mediated degradation of PD-L1 | Promote intrahepatic cholangiocarcinoma | ^8^ |
| miR-145 | 3’-UTR of SPOP | NA | Inhibit SPOP expression | NA | ^9^ |
| miR-1248 | 3’-UTR of SPOP | NA | Inhibit SPOP expression | Promote low-grade gliomas | ^10^ |
| miR-543 | 3’-UTR of SPOP | NA | Inhibit SPOP expression | Promote GC | ^11^ |
| miR-520 | 3’-UTR of SPOP | NA | Inhibit SPOP expression | Inhibit RCC | ^12^ |
| miR-17-5p | 3’-UTR of SPOP | NA | Inhibit SPOP expression | promote CRC | ^13^ |
| miR-372 | 3’-UTR of SPOP | NA | Inhibit SPOP expression | Inhibit RCC, promote CRC | ^12,14^ |
| miR-373 | 3’-UTR of SPOP | NA | Inhibit SPOP expression | Inhibit RCC, promote CRC and oral squamous cell carcinoma | ^12,14,15^ |
| miR-145-5p | 3’-UTR of SPOP | NA | Inhibit SPOP expression | Promote esophageal adenocarcinoma | ^16^ |
| miR-873-5p | 3’-UTR of HMOX1 | NA | promote HIF-1α-mediated SPOP transcription | Inhibit glioblastoma | ^17^ |
| CDK1 | NA | phosphorylation | Promote SPOP expression | Inhibit PCa | ^18^ |
| CDK4 | SPOP S6 | phosphorylation | Inhibit degradation of SPOP | Inhibit immune escape of PCa | ^19^ |
| CDK4 | NA | phosphorylation | Promotes SPOP-mediated degradation of PD-L1 | Inhibit BC | ^20^ |
| ATM | SPOP S119 | phosphorylation | Promote SPOP-mediated ubiquitination of 53BP1 and HIPK2 | Promote DNA damage repair | ^21,22^ |
| ATM | SPOP S119 | phosphorylation | Inhibit SPOP-mediated degradation of MCM3 | Promote DNA damage repair | ^23^ |
| CK1 | SBC of ASCT2 | phosphorylation | Promotes SPOP-mediated degradation of ASCT2 | Involved in glutamine metabolism | ^24^ |
| CK1 | SBC of PDK1 | phosphorylation | Promotes SPOP-mediated degradation of PDK1 | Inhibit cancer | ^25^ |
| CK1 | SBC of FLI1 | phosphorylation | Promotes SPOP-mediated degradation of FLI1 | Inhibit Ewing sarcoma | ^26^ |
| GSK3β | SBC of PDK1 | phosphorylation | Promotes SPOP-mediated degradation of PDK1 | Inhibit cancer | ^25^ |
| LIMK2 | SPOP S59, S171 and S226 | phosphorylation | Promote SPOP degradation | Promote PCa | ^27^ |
| AURKA | SPOP S33, T56 and S105 | phosphorylation | Promote SPOP degradation | Promote PCa | ^28^ |
| GRK2 | SPOP S222 | phosphorylation | Promote SPOP degradation | Involved in glutamine metabolism | ^24^ |
| FZR1 | NA | ubiquitination | Promote degradation of SPOP | Promote immune escape of PCa | ^19^ |
| Dzip1 | NA | ubiquitination | inhibit degradation of SPOP | ensure embryo development | ^29^ |
| MDM2 | SPOP | ubiquitination | Promote SPOP degradation | Inhibit HCC | ^30^ |
| CSN6 | HMGCS1 | ubiquitination | Promote MDM2-mediated SPOP degradation | Promote HCC | ^30^ |
| Snail | BTB domain of SPOP | ubiquitination | Promote SPOP degradation | Promote PCa | ^31^ |
| SERPINA3 | NA | NA | Promote SPOP expression | Inhibit LC | ^32^ |
| GPER1 | NA | NA | Promote SPOP expression | Inhibit BC | ^33^ |
| Gli2 | Rpb3/Rpb7 | NA | Promotes SPOP-mediated degradation of Rpb3/Rpb7 | Inhibit cancer | ^34^ |
| GPR30 | SPOP | NA | Promotes SPOP-mediated degradation of NLRP3 | Inhibit inflammation | ^35^ |
| GPR64 | NA | NA | Promotes SPOP-mediated degradation of RIF1 | Promote Ewing sarcoma | ^36^ |
| p38, PI5P | NA | NA | Promote SPOP activity | Signal transduction of phosphoinositide | ^37^ |
| ALDH2 | 260-290aa of PD-L1 | NA | Inhibit SPOP-mediated degradation of PD-L1 | Promote CRC | ^38^ |
| RIG-I | 239-259aa of PD-L1 | NA | Inhibit SPOP-mediated degradation of PD-L1 | Promote CRC | ^39^ |
| TMEM160 | PD-L1 | NA | Inhibit SPOP-mediated degradation of PD-L1 | Promote CRC | ^40^ |
| MSN | PD-L1 | NA | Inhibit SPOP-mediated degradation of PD-L1 | Promote BC | ^41^ |
| LINC01638 | 144-240aa of c-Myc | NA | Inhibit SPOP-mediated degradation of c-Myc | Promote BC | ^42^ |
| circECE1 | 144-240aa of c-Myc | NA | Inhibit SPOP-mediated degradation of c-Myc | Promote osteosarcoma | ^43^ |
| circXRN2 | 279-525aa of LATS1 | NA | Inhibit SPOP-mediated degradation of LATS1 | Promote bladder cancer | ^44^ |
| Sufu | 961aa-end of Gli2, 1-477aa and 1051-1328aa of Gli3 | NA | Inhibit SPOP-mediated degradation of Gli2 and Gli3 | ensure embryo development | ^45^ |
| SHP | 301-371aa of Gli3 | NA | Inhibit SPOP-mediated degradation of Gli3 | Response to endoplasmic reticulum stress | ^46^ |
| Pin1 | Nanog S65 and S71 | phosphorylation | Inhibit SPOP-mediated degradation of Nanog | Promote PCa | ^47^ |
| EGF/MEK/ERK | ILF3 S382 | phosphorylation | Inhibit SPOP-mediated degradation of ILF3 | Promote CRC | ^48^ |
| SGK3 | 17βHSD4 S318 | phosphorylation | Inhibit SPOP-mediated ubiquitination of 17βHSD4 | Promote PCa | ^49^ |
| ERK1/2 | PrLZ Ser40 | phosphorylation | Inhibit SPOP-mediated degradation of PrLZ | Promote PCa | ^50^ |
| TRIM28 | TRIM24 | NA | Inhibit SPOP-mediated degradation of TRIM24 | Promote PCa | ^51^ |
| TRABID | 53BP1 | NA | Inhibit SPOP-mediated ubiquitination of 53BP1 | improve the sensitivity of PCa to PARP inhibitor | ^52^ |
| BCLAF1 | MATH domain | NA | Inhibit SPOP-mediated degradation of PD-L1 | Promote HCC | ^53^ |
| G3BP1 | MATH domain | NA | Inhibit SPOP-mediated degradation of AR and ERα | Promote PCa and EC | ^54,55^ |
| CDK2 | MATH domain | NA | Inhibit SPOP-mediated degradation of cyclin E1 | Promote PCa | ^56^ |
| TLR4 | SPOP | NA | Promote SPOP translocation from the nucleus to the cytoplasm | Inhibit inflammation | ^57^ |
| OGT | SPOP S96 | O-GlcNAcylation | promote the nuclear localization of SPOP | Promote HCC | ^58^ |
| SNX6 | Cullin3 | NA | Inhibit SPOP-mediated degradation of PD-L1 | Promote immune escape | ^59^ |
| SPOPL | BTB domain | NA | Inhibit SPOP | NA | ^60^ |

Supplement Table 2. The downstream regulator of SPOP.

| Downstream | Binding site of downstream | Binding site of SPOP | Type of ubiquitin | Degradation or not | Effects | Reference |
| --- | --- | --- | --- | --- | --- | --- |
| AR | 203-EGSSS-207, 645-ASSTT-649 | MATH domain | NA | YES | Inhibit PCa | ^61,62^ |
| c-Myc | 185-VCSTS-189, 261-PTTSS-265 | MATH domain | NA | YES | Inhibit PCa, BC | ^42,63^ |
| ERG | 42-ASSSS-46 | MATH domain | NA | YES | Inhibit PCa | ^64,65^ |
| SRC-3 | 99-DVSST-103 | MATH and BTB domain | NA | YES | Inhibit PCa | ^66^ |
| DDIT3/CHOP | 97-TSTS-100 | MATH domain | NA | YES | Inhibit PCa | ^67^ |
| DEK | 170-ADSTT-174 | MATH domain | NA | YES | Inhibit PCa | ^68^ |
| CDC20 | 61-GKSSS-65 | MATH domain | NA | YES | Inhibit PCa | ^69^ |
| EgIN2 | 17-PGSSS-21, 67-ATSTT-71 | MATH domain | NA | YES | Inhibit PCa | ^70^ |
| PD-L1 | 283-290aa | MATH domain | NA | YES | Inhibit PCa | ^19^ |
| ATF2 | 142-ESTTS-146, 175-LLTSS-179, 192-PTSST-196, 234-SITSS-238, 318-ATSTT-323, 336-TQSTS-340, 456-SVSTS-460, 462-GVSST-466 | MATH domain | NA | YES | Inhibit PCa | ^71^ |
| Cyclin E1 | cyclin domain | MATH domain | NA | YES | Inhibit PCa | ^56^ |
| GLP | 645-ADTTS-649, 667-ADTTT-671 | MATH domain | NA | YES | Inhibit PCa | ^72^ |
| ELK3 | 129-LRSTS-133 | MATH domain | NA | YES | Inhibit PCa | ^73^ |
| Gli3 | N- and C-terminal regions | MATH domain | NA | YES | Inhibit PCa, LC | ^45,74,75^ |
| TRIM24 | NA | MATH domain | NA | YES | Inhibit PCa | ^68^ |
| FASN | 160-ACSSS-164, 1715-LDSTS-1719, 2251-EGSTT-2255 | BTB domain | NA | YES | Inhibit PCa | ^76^ |
| BRD2 | 287-ADTTT-291 | MATH and BTB domain | NA | YES | Inhibit PCa, EC, CRC | ^77-81^ |
| BRD3 | 250-ADTTT-254 | MATH and BTB domain | NA | YES | Inhibit PCa, EC, CRC | ^77-81^ |
| BRD4 | 296-ADTTT-300 | MATH and BTB domain | NA | YES | Inhibit PCa, EC, CRC | ^77-81^ |
| NANOG | 66-PDSST-70 | MATH and BTB domain | NA | YES | Inhibit PCa | ^82^ |
| Caprin1 | 435-VSSTS-439 | MATH and BTB domain | NA | YES | Inhibit PCa | ^83^ |
| PDK1 | VSSSSSS | MATH and BTB domain | NA | YES | Inhibit PCa | ^25^ |
| PrLZ | 30-42aa | MATH and BTB domain | K48 | YES | Inhibit PCa | ^50^ |
| LPR5 | 1481-ASSSS-1485 | NA | NA | YES | Inhibit PCa | ^84^ |
| CDCA5 | 121-AESSS-127 | NA | NA | YES | Inhibit PCa | ^85^ |
| STING | 317-ADDSS-332, 352-PSTST-356 | NA | NA | YES | Inhibit PCa | ^86^ |
| LIMK2 | NA | NA | NA | YES | Inhibit PCa | ^27^ |
| MCP-1 | NA | NA | NA | YES | Inhibit PCa | ^87^ |
| ITCH | NA | NA | NA | YES | Inhibit PCa | ^88^ |
| HnRNPK | NA | NA | NA | YES | Inhibit PCa | ^89^ |
| 17βHSD4 | 315-RATST-319 | MATH domain | K27, k29 | NO | Inhibit PCa | ^49^ |
| p62/SQSTM1 | 273-PESSS-277 | MATH domain | K6, K27, K29 | NO | Inhibit PCa | ^90^ |
| ASCT2 | 349-GTSSS-353 | MATH domain | K48 | YES | Inhibit BC | ^24^ |
| SRC3 | N-terminus N390 | MATH domain | NA | YES | Inhibit BC | ^91^ |
| ERα | 431-TSS-433, 463-SST-465, 573-STSS-576 | MATH domain | NA | YES | Inhibit BC, EC | ^33,92^ |
| BRMS1 | 189-GSSRS-193 | NA | NA | YES | Inhibit BC | ^93^ |
| PR | NA | MATH domain | NA | YES | Inhibit BC | ^94^ |
| Sec13 | NA | NA | K63 | NO | Inhibit BC | ^95^ |
| IRF1 | 208-PDSTS-212 | MATH domain | NA | YES | Inhibit EC | ^96^ |
| ZBTB3 | 196-LSSTS-200, 272-PSSST-276 | MATH domain | NA | YES | Inhibit EC | ^97^ |
| IRF2BP2 | 447-VHSTT-451 | MATH domain | K48 | YES | Inhibit HCC | ^98^ |
| CREB5 | 138-PQSSS-142, 489-VGSST-493 | MATH domain | K63 | NO | Inhibit HCC | ^99^ |
| HMGCS1 | 143-IESSS-147 | NA | K48 | YES | Inhibit HCC | ^30^ |
| SLC7A1 | NA | NA | NA | YES | Inhibit HCC | ^100^ |
| Nogo-B | 8-LVSSS-14 | MATH domain | NA | YES | Promote HCC | ^58^ |
| HDAC6 | 843-GPSSS-847 | MATH domain | NA | YES | Inhibit CRC | ^101^ |
| ILF3 | 360-PPSTT-364 | MATH and BTB domain | NA | YES | Inhibit CRC | ^48^ |
| DHX9 | 341-PWTSS-345 | NA | NA | YES | Inhibit choriocarcinoma | ^102^ |
| KIF23 | NA | NA | NA | YES | Inhibit choriocarcinoma | ^103^ |
| DPPA2 | 44-VSSTS-48 | MATH domain | NA | YES | Inhibit testicular germ cell tumor | ^104^ |
| SIRT2 | NA | MATH and BTB domain | NA | YES | Inhibit LC | ^105^ |
| FADD | NA | NA | NA | YES | Inhibit LC and inflammatory | ^106,107^ |
| TIAM1 | NA | MATH domain | NA | YES | Inhibit GC | ^108^ |
| PTEN | 359-ASSST-363 | MATH domain | NA | YES | Promote RCC | ^4^ |
| USP7 | 191-VDSSS-195 | MATH domain | NA | YES | Promote RCC | ^12^ |
| Daxx | 608-VSSTS-612, 680-ADSST-684 | MATH domain | NA | YES | Promote RCC | ^109^ |
| Gli2 | C-terminal region | MATH domain | NA | YES | Promote RCC, inhibit GC | ^45,110^ |
| LATS1 | 332-MQSSS-336, 434-PQSSS-438 | NA | NA | YES | Promote RCC | ^111^ |
| c-Jun | NA | NA | NA | NO | Promote RCC | ^112^ |
| TCF4 | NA | NA | NA | NO | Promote RCC | ^113^ |
| RIF1 | 924-VSSSS-928 | NA | NA | YES | Promote Ewing sarcoma | ^36^ |
| DRAK1 | NA | NA | K48 | YES | Promote cervical cancer | ^114^ |
| p-GSK3 | NA | NA | NA | NO | Promote ovarian cancer | ^115^ |
| MyD88 | 146-VDSSV-150 | MATH domain | K6, K11 | NO | Inhibit DLBCL | ^116^ |
| MyD88 | TIR domain | MATH domain | NA | NO | Inhibit inflammatory | ^57^ |
| MyD88 | INT domain | MATH domain | K48 | YES | Inhibit innate immune response | ^117^ |
| EV71-2Apro | 42-VSSTT-46 | MATH domain | K48 | YES | Inhibit enterovirus 71 | ^118^ |
| MAVS | TM domain | MATH domain | K48 | YES | Inhibit spring viremia of carp virus | ^119^ |
| HNF1α | dimer domain | MATH domain | NA | YES | Inhibit HBV infection | ^120^ |
| NLRP3 | NA | NA | K48 | YES | Inhibit inflammatory | ^121^ |
| SETD2 | 1373-SSNS-1376 | MATH domain | K48 | YES | Involve in alternative splicing events | ^122^ |
| MacroH2A1 | 285-ADSST-289 | MATH domain | NA | NO | Stable X chromosome | ^123^ |
| BMI-1 | central and C-terminal parts | MATH domain | NA | NO | Stable X chromosome | ^124^ |
| HIPK2 | 97-ASSTS-101, 863-ASSTT-867 | MATH domain | K27, K29, K33, K48, K63 | NO | Promote genomic stability | ^22^ |
| 53BP1 | 1641-ASSSS-1645 | MATH domain | K29 | NO | Promote genomic stability | ^21^ |
| Geminin | 200-VSSST-204 | MATH domain | K27 | NO | Inhibit replication catastrophe | ^125^ |
| CDT1, CDC6 | NA | NA | NA | NO | Ensure DNA replication | ^126^ |
| Gli3 | 1-700aa | MATH domain | NA | YES | Promote development | ^127^ |
| SENP7 | 201-LSSSS-205 | NA | NA | YES | Promote senescence | ^128^ |
| INF2 | 1144-ADSTS-1148 | MATH domain | K27, K29, K33, K48, K63 | NO | Inhibit mitochondrial fission | ^129^ |
| XBP1 | NA | NA | NA | YES | Involve in endoplasmic reticulum stress | ^46^ |
| Nup153 | 1-330aa | MATH domain | NA | YES | Inhibit the nuclear membrane localization of Mad1 | ^130^ |
| Pdx1 | 229-VTSGE-233, 269-PQPSS-273 | MATH domain | NA | YES | Promote glucose homeostasis | ^131,132^ |
| RACK1 | 94-TGTTT-98, 275-ISTSS-279 | MATH domain | NA | YES | Promote cardiac fibrosis | ^133^ |
| PIPKIIβ | NA | BTB domain | NA | YES | Involve in phosphoinositide signaling | ^37^ |
| hemoglobin | NA | NA | NA | NO | Inhibit hemoglobin | ^134^ |

Supplement Table 3. The abbreviations.

| Full name | Abbreviations |
| --- | --- |
| 17beta-hydroxysteroid dehydrogenase type 4 | 17βHSD4 |
| 53-binding protein 1 | 53BP1 |
| activator protein 1 | AP-1 |
| activating transcription factor 2 | ATF2 |
| the ataxia-telangiectasia mutated | ATM |
| alanine-serine-cysteine transporter 2 | ASCT2 |
| aldehyde dehydrogenase 2 | ALDH2 |
| amyotrophic lateral sclerosis | ALS |
| androgen receptor | AR |
| androgen receptor splice variant 7 | ARv7 |
| aryl hydrocarbon receptor | AhR |
| Ataxia telangiectasia and Rad3-related protein | ATR |
| aurora kinase A | AURKA |
| basic helix-loop-helix leucine zipper | BHLH-ZIP |
| B-cell lymphoma/leukemia 11A | BCL11A |
| B-cell lymphoma-2-associated transcription factor 1 | BCLAF1 |
| breast cancer | BC |
| Breast cancer type 1 susceptibility protein | BRCA1 |
| bromodomain-containing protein | BRD |
| casein kinase 1 | CK1 |
| cell division cycle 6 | CDC6 |
| cell division cycle 25A | CDC25A |
| chromosome 1 | CHR1 |
| checkpoint kinase 1 | CHK1 |
| Cdc10-dependent transcript 1 protein | CDT1 |
| colorectal cancer | CRC |
| COP9 signalosome complex subunit 6 | CSN6 |
| C-terminal Src kinase | CSK |
| Cyclic AMP-responsive element-binding protein 5 | CREB5 |
| cyclic GMP-AMP | cGAMP |
| cyclin-dependent kinase 1 | CDK |
| death-associated protein kinase-related apoptosis-inducing kinase 1 | DRAK1 |
| DAZ-interacting protein | Dzip1 |
| death domain-associated protein 6 | Daxx |
| Diffuse large B-cell lymphoma | DLBCL |
| dipeptide repeat protein | DPR |
| disease free interval | DFI |
| disease specific survival | DSS |
| DNA double-strand break | DSB |
| E2 promoter binding factor 1 | E2F1 |
| E26 transformation-specific (ETS) transcription factor ELK3 | ELK3 |
| endometrial cancer | EC |
| enterovirus 71 | EV71 |
| estrogen receptor alpha | ERα |
| ETS-related gene | ERG |
| FAS-associating death domain-containing protein | FADD |
| fizzy-related protein homolog 1 | FZR1 |
| frontotemporal dementia | FTD |
| GAP activity towards Rags 2 | GATOR2 |
| Ras-GTPase-activating protein binding protein 1 | G3BP1 |
| gastric carcinoma | GC |
| gene set enrichment analysis | GSEA |
| glycogen synthase kinase 3β | GSK3β |
| G-protein coupled estrogen receptor 1 | GPER1 |
| G-protein coupled receptor | GPR |
| G-protein coupled receptor kinase 2 | GRK2 |
| trimethylated histone H3 | H3K9me3 |
| Heme oxygenase 1 | HMOX1 |
| hepatocellular carcinoma | HCC |
| hepatitis C virus | HCV |
| hepatocyte nuclear factor 1alpha | HNF1α |
| heterochromatin protein 1 gamma | HP1γ |
| histone deacetylase 1 | HDAC1 |
| homeodomain-interacting protein kinase 2 | HIPK2 |
| hydroxymethylglutaryl-CoA synthase 1 | HMGCS1 |
| hypoxia inducible factor-1α | HIF-1α |
| interferon regulatory factor | IRF |
| interferon regulatory factor 2-binding protein 2 | IRF2BP2 |
| interleukin enhancer-binding factor 3 | ILF3 |
| interleukin-1 receptor-associated kinase 4 | IRAK4 |
| inverted formin-2 | INF2 |
| kinesin family member 23 | KIF23 |
| LIM kinase-2 | LIMK2 |
| Lipocalin-2 | LCN2 |
| lipoprotein receptor-related protein 5 | LPR5 |
| liquid-liquid phase separation | LLPS |
| minichromosome maintenance complex component 3 | MCM3 |
| mitochondrial antiviral signaling protein | MAVS |
| mitotic arrest deficiency 1 | Mad1 |
| Moesin | MSN |
| Monocyte chemoattractant protein-1 | MCP-1 |
| Murine double minute 2 | MDM2 |
| myeloid differentiation factor-88 | MyD88 |
| neurite outgrowth inhibitor-B | Nogo-B |
| non-small cell lung cancer | NSCLC |
| nonstructural 4B protein | NS4B |
| nucleoporin protein 153 | Nup153 |
| O-GlcNAcylated by O-GlcNAc transferase | OGT |
| overall survival | OS |
| pathogen-associated molecular patterns | PAMPs |
| 3-phosphoinositide-dependent protein kinase-1 | PDK1 |
| pattern recognition receptor | PRR |
| peptidyl-prolyl isomerase | Pin1 |
| peripheral T-cell lymphoma | PTCL |
| phosphatase and Tensin homolog deleted on chromosome ten | PTEN |
| phosphatidylinositol 5 phosphate | PI5P |
| programmed cell death 1 ligand 1 | PD-L1 |
| prostate cancer | PCa |
| Prostate leucine zipper | PrLZ |
| PTEN-induced putative kinase 1 | PINK1 |
| radioprotective 105 kDa protein | RP105 |
| Rap1-interacting factor 1 homolog | RIF1 |
| Receptor of activated protein C kinase 1 | RACK1 |
| renal clear cell carcinoma | RCC |
| Reprimo | RPRM |
| retinoblastoma tumor suppressor gene | RB |
| retinoic acid-inducible gene 1 protein | RIG-I |
| retinoid X receptor alpha | RXRA |
| Rho-associated protein kinase | ROCK |
| RING finger protein 125 | RNF125 |
| SUMO-specific protease 7 | SENP7 |
| serine protease inhibitor clade A member 3 | SERPINA3 |
| serum & glucocorticoid inducible kinase 3 | SGK3 |
| SET domain-containing protein 2 | SETD2 |
| small heterodimer partner | SHP |
| sorting nexin-6 | SNX6 |
| specificity protein 1 | SP1 |
| speckle-type POZ protein | SPOP |
| Speckle-type POZ protein-like | SPOPL |
| sphingomyelin phosphodiesterase 1 | SMPD1 |
| SPOP-binding consensus | SBC |
| suppressor of fused homolog | Sufu |
| TANK-binding kinase 1 | TBK1 |
| large tumor suppressor 1 | LATS1 |
| The Replication Protein A | RPA |
| T-lymphoma invasion and metastasis 1 | TIAM1 |
| Toll-like receptor | TLR |
| TRAF-binding domain-containing protein | TRABID |
| transcription factor 4 | TCF4 |
| transmembrane protein 160 | TMEM160 |
| tripartite motif-containing protein 28 | TRIM28 |
| Tumor necrosis factor | TNF |
| tumor necrosis factor-receptor associated factor | TRAF |
| Twist-related protein 1 | TWIST1 |
| ubiquitin-specific peptidase 7 | USP7 |
| ubiquitin-proteasome system | UPS |
| X-box binding protein 1 | XBP1 |
| Zuojinwan | ZJW |

1	Yao, S. *et al.* Speckle-type POZ protein functions as a tumor suppressor in non-small cell lung cancer due to DNA methylation. *Cancer Cell Int* **18**, 213 (2018). <https://doi.org:10.1186/s12935-018-0711-z>

2	Zhi, X. *et al.* Silencing speckle-type POZ protein by promoter hypermethylation decreases cell apoptosis through upregulating Hedgehog signaling pathway in colorectal cancer. *Cell death & disease* **7**, e2569 (2016). <https://doi.org:10.1038/cddis.2016.435>

3	Bernasocchi, T. *et al.* Dual functions of SPOP and ERG dictate androgen therapy responses in prostate cancer. *Nature communications* **12**, 734 (2021). <https://doi.org:10.1038/s41467-020-20820-x>

4	Li, G. *et al.* SPOP promotes tumorigenesis by acting as a key regulatory hub in kidney cancer. *Cancer Cell* **25**, 455-468 (2014). <https://doi.org:10.1016/j.ccr.2014.02.007>

5	Yuan, D. *et al.* Hypoxia-induced SPOP attenuates the mobility of trophoblast cells through inhibition of the PI3K/AKT/GSK3β pathway. *Cell biology international* **45**, 599-611 (2021). <https://doi.org:10.1002/cbin.11501>

6	Jiao, C. *et al.* TGF-β signaling regulates SPOP expression and promotes prostate cancer cell stemness. *Aging* **12**, 7747-7760 (2020). <https://doi.org:10.18632/aging.103085>

7	Wang, F. *et al.* LncRNA ADAMTS9-AS2 suppresses the proliferation of gastric cancer cells and the tumorigenicity of cancer stem cells through regulating SPOP. *J Cell Mol Med* **24**, 4830-4838 (2020). <https://doi.org:10.1111/jcmm.15161>

8	Li, J. *et al.* N6-methyladenosine-modified circSLCO1B3 promotes intrahepatic cholangiocarcinoma progression via regulating HOXC8 and PD-L1. *Journal of experimental & clinical cancer research : CR* **43**, 119 (2024). <https://doi.org:10.1186/s13046-024-03006-x>

9	Huang, C. J., Chen, H. Y., Lin, W. Y. & Choo, K. B. Differential expression of speckled POZ protein, SPOP: putative regulation by miR-145. *J Biosci* **39**, 401-413 (2014). <https://doi.org:10.1007/s12038-014-9432-1>

10	Catanzaro, G. *et al.* MiR-1248: a new prognostic biomarker able to identify supratentorial hemispheric pediatric low-grade gliomas patients associated with progression. *Biomarker research* **10**, 44 (2022). <https://doi.org:10.1186/s40364-022-00389-x>

11	Xu, J., Wang, F., Wang, X., He, Z. & Zhu, X. miRNA-543 promotes cell migration and invasion by targeting SPOP in gastric cancer. *OncoTargets and therapy* **11**, 5075-5082 (2018). <https://doi.org:10.2147/ott.S161316>

12	Ding, M. *et al.* The E2F1-miR-520/372/373-SPOP Axis Modulates Progression of Renal Carcinoma. *Cancer Res* **78**, 6771-6784 (2018). <https://doi.org:10.1158/0008-5472.Can-18-1662>

13	Sun, W. *et al.* Tumor stem cell-derived exosomal microRNA-17-5p inhibits anti-tumor immunity in colorectal cancer via targeting SPOP and overexpressing PD-L1. *Cell death discovery* **8**, 223 (2022). <https://doi.org:10.1038/s41420-022-00919-4>

14	Wang, L. Q. *et al.* miR-372 and miR-373 enhance the stemness of colorectal cancer cells by repressing differentiation signaling pathways. *Molecular oncology* **12**, 1949-1964 (2018). <https://doi.org:10.1002/1878-0261.12376>

15	Zhang, X. J., Jin, Y., Song, J. L. & Deng, F. MiR-373 promotes proliferation and metastasis of oral squamous cell carcinoma by targeting SPOP. *European review for medical and pharmacological sciences* **23**, 5270-5276 (2019). <https://doi.org:10.26355/eurrev_201906_18193>

16	Zhang, L. *et al.* c-Myb facilitates immune escape of esophageal adenocarcinoma cells through the miR-145-5p/SPOP/PD-L1 axis. *Clinical and translational medicine* **11**, e464 (2021). <https://doi.org:10.1002/ctm2.464>

17	Zhang, X. *et al.* Tumor-suppressive function and mechanism of miR-873-5p in glioblastoma: evidence based on bioinformatics analysis and experimental validation. *Aging* **15**, 5412-5425 (2023). <https://doi.org:10.18632/aging.204800>

18	Tang, Z. *et al.* ATR Inhibition Induces CDK1-SPOP Signaling and Enhances Anti-PD-L1 Cytotoxicity in Prostate Cancer. *Clin Cancer Res* **27**, 4898-4909 (2021). <https://doi.org:10.1158/1078-0432.Ccr-21-1010>

19	Zhang, J. *et al.* Cyclin D-CDK4 kinase destabilizes PD-L1 via cullin 3-SPOP to control cancer immune surveillance. *Nature* **553**, 91-95 (2018). <https://doi.org:10.1038/nature25015>

20	Shrestha, M., Wang, D. Y., Ben-David, Y. & Zacksenhaus, E. CDK4/6 inhibitors and the pRB-E2F1 axis suppress PVR and PD-L1 expression in triple-negative breast cancer. *Oncogenesis* **12**, 29 (2023). <https://doi.org:10.1038/s41389-023-00475-1>

21	Wang, D. *et al.* ATM-phosphorylated SPOP contributes to 53BP1 exclusion from chromatin during DNA replication. *Science advances* **7** (2021). <https://doi.org:10.1126/sciadv.abd9208>

22	Jin, X. *et al.* Prostate cancer-associated SPOP mutations lead to genomic instability through disruption of the SPOP-HIPK2 axis. *Nucleic Acids Res* **49**, 6788-6803 (2021). <https://doi.org:10.1093/nar/gkab489>

23	Xiao, M. *et al.* A disease-relevant mutation of SPOP highlights functional significance of ATM-mediated DNA damage response. *Signal Transduction and Targeted Therapy* **6** (2021). <https://doi.org:10.1038/s41392-020-00381-7>

24	Zhou, Q. *et al.* Neddylation inhibition induces glutamine uptake and metabolism by targeting CRL3(SPOP) E3 ligase in cancer cells. *Nature communications* **13**, 3034 (2022). <https://doi.org:10.1038/s41467-022-30559-2>

25	Jiang, Q. *et al.* SPOP-mediated ubiquitination and degradation of PDK1 suppresses AKT kinase activity and oncogenic functions. *Mol Cancer* **20**, 100 (2021). <https://doi.org:10.1186/s12943-021-01397-5>

26	Su, S. *et al.* SPOP and OTUD7A Control EWS-FLI1 Protein Stability to Govern Ewing Sarcoma Growth. *Advanced science (Weinheim, Baden-Wurttemberg, Germany)* **8**, e2004846 (2021). <https://doi.org:10.1002/advs.202004846>

27	Nikhil, K., Haymour, H. S., Kamra, M. & Shah, K. Phosphorylation-dependent regulation of SPOP by LIMK2 promotes castration-resistant prostate cancer. *British journal of cancer* **124**, 995-1008 (2021). <https://doi.org:10.1038/s41416-020-01197-6>

28	Nikhil, K., Kamra, M., Raza, A., Haymour, H. S. & Shah, K. Molecular Interplay between AURKA and SPOP Dictates CRPC Pathogenesis via Androgen Receptor. *Cancers* **12** (2020). <https://doi.org:10.3390/cancers12113247>

29	Schwend, T. *et al.* Stabilization of speckle-type POZ protein (Spop) by Daz interacting protein 1 (Dzip1) is essential for Gli turnover and the proper output of Hedgehog signaling. *The Journal of biological chemistry* **288**, 32809-32820 (2013). <https://doi.org:10.1074/jbc.M113.512962>

30	Li, K. *et al.* CSN6-SPOP-HMGCS1 Axis Promotes Hepatocellular Carcinoma Progression via YAP1 Activation. *Advanced science (Weinheim, Baden-Wurttemberg, Germany)* **11**, e2306827 (2024). <https://doi.org:10.1002/advs.202306827>

31	Lv, W. *et al.* Snail promotes prostate cancer migration by facilitating SPOP ubiquitination and degradation. *Biochemical and biophysical research communications* **529**, 799-804 (2020). <https://doi.org:10.1016/j.bbrc.2020.05.187>

32	Jin, Y. *et al.* Overexpression of SERPINA3 suppresses tumor progression by modulating SPOP/NF‑κB in lung cancer. *Int J Oncol* **63** (2023). <https://doi.org:10.3892/ijo.2023.5544>

33	Zhang, N. *et al.* The GPER1/SPOP axis mediates ubiquitination-dependent degradation of ERα to inhibit the growth of breast cancer induced by oestrogen. *Cancer letters* **498**, 54-69 (2021). <https://doi.org:10.1016/j.canlet.2020.10.019>

34	Gao, Y. *et al.* HIB/SPOP inhibits Ci/Gli-mediated tumorigenesis by modulating the RNA Polymerase II components stabilities. *iScience* **26**, 107334 (2023). <https://doi.org:10.1016/j.isci.2023.107334>

35	Peng, J., He, J., Lin, L., Li, Y. & Xia, Y. Neural Stem Cell Extracellular Vesicles Carrying YBX1 Inhibited Neuronal Pyroptosis Through Increasing m6A-modified GPR30 Stability and Expression in Ischemic Stroke. *Transl Stroke Res* (2023). <https://doi.org:10.1007/s12975-023-01210-z>

36	Suvarna, K. *et al.* Ceramide-induced cleavage of GPR64 intracellular domain drives Ewing sarcoma. *Cell reports* **43**, 114497 (2024). <https://doi.org:10.1016/j.celrep.2024.114497>

37	Bunce, M. W., Boronenkov, I. V. & Anderson, R. A. Coordinated activation of the nuclear ubiquitin ligase Cul3-SPOP by the generation of phosphatidylinositol 5-phosphate. *The Journal of biological chemistry* **283**, 8678-8686 (2008). <https://doi.org:10.1074/jbc.M710222200>

38	Zhang, H. *et al.* Aldehyde Dehydrogenase 2 Mediates Alcohol-Induced Colorectal Cancer Immune Escape through Stabilizing PD-L1 Expression. *Advanced science (Weinheim, Baden-Wurttemberg, Germany)* **8**, 2003404 (2021). <https://doi.org:10.1002/advs.202003404>

39	Zhang, Y. *et al.* RIG-I promotes immune evasion of colon cancer by modulating PD-L1 ubiquitination. *Journal for immunotherapy of cancer* **11** (2023). <https://doi.org:10.1136/jitc-2023-007313>

40	Dai, X. *et al.* TMEM160 promotes tumor immune evasion and radiotherapy resistance via PD-L1 binding in colorectal cancer. *Cell communication and signaling : CCS* **22**, 168 (2024). <https://doi.org:10.1186/s12964-024-01541-w>

41	Meng, F., Su, Y. & Xu, B. Rho-associated protein kinase-dependent moesin phosphorylation is required for PD-L1 stabilization in breast cancer. *Molecular oncology* **14**, 2701-2712 (2020). <https://doi.org:10.1002/1878-0261.12804>

42	Luo, L. *et al.* LINC01638 lncRNA activates MTDH-Twist1 signaling by preventing SPOP-mediated c-Myc degradation in triple-negative breast cancer. *Oncogene* **37**, 6166-6179 (2018). <https://doi.org:10.1038/s41388-018-0396-8>

43	Shen, S. *et al.* CircECE1 activates energy metabolism in osteosarcoma by stabilizing c-Myc. *Molecular cancer* **19**, 151 (2020). <https://doi.org:10.1186/s12943-020-01269-4>

44	Xie, B. *et al.* CircXRN2 suppresses tumor progression driven by histone lactylation through activating the Hippo pathway in human bladder cancer. *Mol Cancer* **22**, 151 (2023). <https://doi.org:10.1186/s12943-023-01856-1>

45	Wang, C., Pan, Y. & Wang, B. Suppressor of fused and Spop regulate the stability, processing and function of Gli2 and Gli3 full-length activators but not their repressors. *Development* **137**, 2001-2009 (2010). <https://doi.org:10.1242/dev.052126>

46	Sun, S., Kelekar, S., Kliewer, S. A. & Mangelsdorf, D. J. The orphan nuclear receptor SHP regulates ER stress response by inhibiting XBP1s degradation. *Genes & development* **33**, 1083-1094 (2019). <https://doi.org:10.1101/gad.326868.119>

47	Zhang, J. *et al.* SPOP Promotes Nanog Destruction to Suppress Stem Cell Traits and Prostate Cancer Progression. *Developmental cell* **48**, 329-344.e325 (2019). <https://doi.org:10.1016/j.devcel.2018.11.035>

48	Li, K. *et al.* ILF3 is a substrate of SPOP for regulating serine biosynthesis in colorectal cancer. *Cell research* **30**, 163-178 (2020). <https://doi.org:10.1038/s41422-019-0257-1>

49	Shi, L. *et al.* Mutated SPOP E3 Ligase Promotes 17βHSD4 Protein Degradation to Drive Androgenesis and Prostate Cancer Progression. *Cancer Res* **81**, 3593-3606 (2021). <https://doi.org:10.1158/0008-5472.Can-20-3258>

50	Fan, Y. *et al.* ERK1/2 inhibits Cullin 3/SPOP-mediated PrLZ ubiquitination and degradation to modulate prostate cancer progression. *Cell death and differentiation* **29**, 1611-1624 (2022). <https://doi.org:10.1038/s41418-022-00951-y>

51	Fong, K. W., Zhao, J. C., Song, B., Zheng, B. & Yu, J. TRIM28 protects TRIM24 from SPOP-mediated degradation and promotes prostate cancer progression. *Nature communications* **9**, 5007 (2018). <https://doi.org:10.1038/s41467-018-07475-5>

52	Ma, J. *et al.* TRABID overexpression enables synthetic lethality to PARP inhibitor via prolonging 53BP1 retention at double-strand breaks. *Nature communications* **14**, 1810 (2023). <https://doi.org:10.1038/s41467-023-37499-5>

53	Yu, Z. *et al.* BCLAF1 binds SPOP to stabilize PD-L1 and promotes the development and immune escape of hepatocellular carcinoma. *Cellular and molecular life sciences : CMLS* **81**, 82 (2024). <https://doi.org:10.1007/s00018-024-05144-z>

54	Ge, Y. *et al.* Endometrial cancer (EC) derived G3BP1 overexpression and mutant promote EC tumorigenesis and metastasis via SPOP/ERα axis. *Cell communication and signaling : CCS* **21**, 303 (2023). <https://doi.org:10.1186/s12964-023-01342-7>

55	Mukhopadhyay, C. *et al.* G3BP1 inhibits Cul3(SPOP) to amplify AR signaling and promote prostate cancer. *Nat Commun* **12**, 6662 (2021). <https://doi.org:10.1038/s41467-021-27024-x>

56	Ju, L. G. *et al.* SPOP suppresses prostate cancer through regulation of CYCLIN E1 stability. *Cell Death Differ* **26**, 1156-1168 (2019). <https://doi.org:10.1038/s41418-018-0198-0>

57	Hu, Y. H. *et al.* SPOP negatively regulates Toll-like receptor-induced inflammation by disrupting MyD88 self-association. *Cellular & molecular immunology* **18**, 1708-1717 (2021). <https://doi.org:10.1038/s41423-020-0411-1>

58	Zhou, P. *et al.* O-GlcNAcylation of SPOP promotes carcinogenesis in hepatocellular carcinoma. *Oncogene* **42**, 725-736 (2023). <https://doi.org:10.1038/s41388-022-02589-z>

59	Ghosh, C. *et al.* Sorting nexin 6 interacts with Cullin3 and regulates programmed death ligand 1 expression. *FEBS Lett* **595**, 2558-2569 (2021). <https://doi.org:10.1002/1873-3468.14191>

60	Errington, W. J. *et al.* Adaptor protein self-assembly drives the control of a cullin-RING ubiquitin ligase. *Structure* **20**, 1141-1153 (2012). <https://doi.org:10.1016/j.str.2012.04.009>

61	An, J., Wang, C., Deng, Y., Yu, L. & Huang, H. Destruction of full-length androgen receptor by wild-type SPOP, but not prostate-cancer-associated mutants. *Cell reports* **6**, 657-669 (2014). <https://doi.org:10.1016/j.celrep.2014.01.013>

62	Geng, C. *et al.* Androgen receptor is the key transcriptional mediator of the tumor suppressor SPOP in prostate cancer. *Cancer Res* **74**, 5631-5643 (2014). <https://doi.org:10.1158/0008-5472.Can-14-0476>

63	Geng, C. *et al.* SPOP regulates prostate epithelial cell proliferation and promotes ubiquitination and turnover of c-MYC oncoprotein. *Oncogene* **36**, 4767-4777 (2017). <https://doi.org:10.1038/onc.2017.80>

64	Gan, W. *et al.* SPOP Promotes Ubiquitination and Degradation of the ERG Oncoprotein to Suppress Prostate Cancer Progression. *Mol Cell* **59**, 917-930 (2015). <https://doi.org:10.1016/j.molcel.2015.07.026>

65	An, J. *et al.* Truncated ERG Oncoproteins from TMPRSS2-ERG Fusions Are Resistant to SPOP-Mediated Proteasome Degradation. *Mol Cell* **59**, 904-916 (2015). <https://doi.org:10.1016/j.molcel.2015.07.025>

66	Geng, C. *et al.* Prostate cancer-associated mutations in speckle-type POZ protein (SPOP) regulate steroid receptor coactivator 3 protein turnover. *Proc Natl Acad Sci U S A* **110**, 6997-7002 (2013). <https://doi.org:10.1073/pnas.1304502110>

67	Zhang, P. *et al.* Destruction of DDIT3/CHOP protein by wild-type SPOP but not prostate cancer-associated mutants. *Hum Mutat* **35**, 1142-1151 (2014). <https://doi.org:10.1002/humu.22614>

68	Theurillat, J. P. *et al.* Prostate cancer. Ubiquitylome analysis identifies dysregulation of effector substrates in SPOP-mutant prostate cancer. *Science (New York, N.Y.)* **346**, 85-89 (2014). <https://doi.org:10.1126/science.1250255>

69	Wu, F. *et al.* Prostate cancer-associated mutation in SPOP impairs its ability to target Cdc20 for poly-ubiquitination and degradation. *Cancer Lett* **385**, 207-214 (2017). <https://doi.org:10.1016/j.canlet.2016.10.021>

70	Zhang, L. *et al.* Tumor suppressor SPOP ubiquitinates and degrades EglN2 to compromise growth of prostate cancer cells. *Cancer Lett* **390**, 11-20 (2017). <https://doi.org:10.1016/j.canlet.2017.01.003>

71	Bouchard, J. J. *et al.* Cancer Mutations of the Tumor Suppressor SPOP Disrupt the Formation of Active, Phase-Separated Compartments. *Molecular Cell* **72**, 19-+ (2018). <https://doi.org:10.1016/j.molcel.2018.08.027>

72	Zhang, J. *et al.* SPOP mutation induces DNA methylation via stabilizing GLP/G9a. *Nature communications* **12**, 5716 (2021). <https://doi.org:10.1038/s41467-021-25951-3>

73	Lee, C. J. *et al.* ELK3 destabilization by speckle-type POZ protein suppresses prostate cancer progression and docetaxel resistance. *Cell death & disease* **15**, 274 (2024). <https://doi.org:10.1038/s41419-024-06647-0>

74	Burleson, M. *et al.* GLI3 Is Stabilized by SPOP Mutations and Promotes Castration Resistance via Functional Cooperation with Androgen Receptor in Prostate Cancer. *Mol Cancer Res* (2021). <https://doi.org:10.1158/1541-7786.Mcr-21-0108>

75	Liu, X., Liu, J., Zhang, X., Tong, Y. & Gan, X. MiR-520b promotes the progression of non-small cell lung cancer through activating Hedgehog pathway. *Journal of cellular and molecular medicine* **23**, 205-215 (2019). <https://doi.org:10.1111/jcmm.13909>

76	Gang, X. *et al.* Speckle-type POZ protein suppresses lipid accumulation and prostate cancer growth by stabilizing fatty acid synthase. *Prostate* **79**, 864-871 (2019). <https://doi.org:10.1002/pros.23793>

77	Zhang, P. *et al.* Intrinsic BET inhibitor resistance in SPOP-mutated prostate cancer is mediated by BET protein stabilization and AKT-mTORC1 activation. *Nature medicine* **23**, 1055-1062 (2017). <https://doi.org:10.1038/nm.4379>

78	Dai, X., Wang, Z. & Wei, W. SPOP-mediated degradation of BRD4 dictates cellular sensitivity to BET inhibitors. *Cell Cycle* **16**, 2326-2329 (2017). <https://doi.org:10.1080/15384101.2017.1388973>

79	Janouskova, H. *et al.* Opposing effects of cancer-type-specific SPOP mutants on BET protein degradation and sensitivity to BET inhibitors. *Nat Med* **23**, 1046-1054 (2017). <https://doi.org:10.1038/nm.4372>

80	Ostertag, M. S., Hutwelker, W., Plettenburg, O., Sattler, M. & Popowicz, G. M. Structural Insights into BET Client Recognition of Endometrial and Prostate Cancer-Associated SPOP Mutants. *J Mol Biol* **431**, 2213-2221 (2019). <https://doi.org:10.1016/j.jmb.2019.04.017>

81	Tong, J. *et al.* BET protein degradation triggers DR5-mediated immunogenic cell death to suppress colorectal cancer and potentiate immune checkpoint blockade. *Oncogene* **40**, 6566-6578 (2021). <https://doi.org:10.1038/s41388-021-02041-8>

82	Wang, X. *et al.* AMPK Promotes SPOP-Mediated NANOG Degradation to Regulate Prostate Cancer Cell Stemness. *Dev Cell* **48**, 345-360.e347 (2019). <https://doi.org:10.1016/j.devcel.2018.11.033>

83	Shi, Q. *et al.* Prostate Cancer-associated SPOP mutations enhance cancer cell survival and docetaxel resistance by upregulating Caprin1-dependent stress granule assembly. *Mol Cancer* **18**, 170 (2019). <https://doi.org:10.1186/s12943-019-1096-x>

84	Gan, S. *et al.* LRP5 competes for SPOP binding to enhance tumorigenesis mediated by Daxx and PD-L1 in prostate cancer. *Exp Cell Res* **434**, 113857 (2024). <https://doi.org:10.1016/j.yexcr.2023.113857>

85	Luo, Z. *et al.* SPOP promotes CDCA5 degradation to regulate prostate cancer progression via the AKT pathway. *Neoplasia* **23**, 1037-1047 (2021). <https://doi.org:10.1016/j.neo.2021.08.002>

86	Geng, C. *et al.* SPOP Mutations Target STING1 Signaling in Prostate Cancer and Create Therapeutic Vulnerabilities to PARP Inhibitor-Induced Growth Suppression. *Clin Cancer Res* **29**, 4464-4478 (2023). <https://doi.org:10.1158/1078-0432.Ccr-23-1439>

87	Shi, J. *et al.* Loss of Speckle-Type POZ Protein Promotes Prostate Cancer Cell Migration and Invasion Through Upregulation of MCP-1. *Med Sci Monit* **27**, e929199 (2021). <https://doi.org:10.12659/msm.929199>

88	Ma, J. *et al.* The SPOP-ITCH Signaling Axis Protects Against Prostate Cancer Metastasis. *Front Oncol* **11**, 658230 (2021). <https://doi.org:10.3389/fonc.2021.658230>

89	Wu, H. L. *et al.* Transcriptional regulation and ubiquitination-dependent regulation of HnRNPK oncogenic function in prostate tumorigenesis. *Cancer Cell Int* **21**, 641 (2021). <https://doi.org:10.1186/s12935-021-02331-x>

90	Shi, Q. *et al.* SPOP mutations promote p62/SQSTM1-dependent autophagy and Nrf2 activation in prostate cancer. *Cell Death & Differentiation* (2022). <https://doi.org:10.1038/s41418-021-00913-w>

91	Li, C. *et al.* Tumor-suppressor role for the SPOP ubiquitin ligase in signal-dependent proteolysis of the oncogenic co-activator SRC-3/AIB1. *Oncogene* **30**, 4350-4364 (2011). <https://doi.org:10.1038/onc.2011.151>

92	Zhang, P. *et al.* Endometrial cancer-associated mutants of SPOP are defective in regulating estrogen receptor-α protein turnover. *Cell death & disease* **6**, e1687 (2015). <https://doi.org:10.1038/cddis.2015.47>

93	Kim, B. *et al.* Breast cancer metastasis suppressor 1 (BRMS1) is destabilized by the Cul3-SPOP E3 ubiquitin ligase complex. *Biochem Biophys Res Commun* **415**, 720-726 (2011). <https://doi.org:10.1016/j.bbrc.2011.10.154>

94	Gao, K. *et al.* Tumor suppressor SPOP mediates the proteasomal degradation of progesterone receptors (PRs) in breast cancer cells. *American journal of cancer research* **5**, 3210-3220 (2015).

95	Yang, Y. *et al.* SPOP negatively regulates mTORC1 activity by ubiquitinating Sec13. *Cell Signal* **116**, 111060 (2024). <https://doi.org:10.1016/j.cellsig.2024.111060>

96	Gao, K. *et al.* SPOP mutations promote tumor immune escape in endometrial cancer via the IRF1-PD-L1 axis. *Cell Death Differ* **30**, 475-487 (2023). <https://doi.org:10.1038/s41418-022-01097-7>

97	Jin, X. *et al.* SPOP targets oncogenic protein ZBTB3 for destruction to suppress endometrial cancer. *American journal of cancer research* **9**, 2797-2812 (2019).

98	Deng, Y. *et al.* SPOP point mutations regulate substrate preference and affect its function. *Cell Death Dis* **15**, 172 (2024). <https://doi.org:10.1038/s41419-024-06565-1>

99	Gong, D. A. *et al.* SPOP promotes CREB5 ubiquitination to inhibit MET signaling in liver cancer. *Biochimica et biophysica acta. Molecular cell research* **1871**, 119642 (2024). <https://doi.org:10.1016/j.bbamcr.2023.119642>

100	He, W. *et al.* S119N Mutation of the E3 Ubiquitin Ligase SPOP Suppresses SLC7A1 Degradation to Regulate Hepatoblastoma Progression. *Mol Ther Oncolytics* **19**, 149-162 (2020). <https://doi.org:10.1016/j.omto.2020.09.008>

101	Tan, Y. *et al.* Cullin 3SPOP ubiquitin E3 ligase promotes the poly-ubiquitination and degradation of HDAC6. *Oncotarget* **8**, 47890-47901 (2017). <https://doi.org:10.18632/oncotarget.18141>

102	Yuan, D. *et al.* SPOP attenuates migration and invasion of choriocarcinoma cells by promoting DHX9 degradation. *American journal of cancer research* **10**, 2428-2445 (2020).

103	Zhou, C. *et al.* SPOP attenuates the proliferation, invasion, and migration of choriocarcinoma JAR cells by promoting KIF23 degradation. *Oncologie* **26**, 467-477 (2024). <https://doi.org:10.1515/oncologie-2023-0595>

104	Wang, J. *et al.* SPOP suppresses testicular germ cell tumors progression through ubiquitination and degradation of DPPA2. *Biochem Biophys Res Commun* **557**, 55-61 (2021). <https://doi.org:10.1016/j.bbrc.2021.04.002>

105	Luo, J. *et al.* SPOP promotes SIRT2 degradation and suppresses non-small cell lung cancer cell growth. *Biochem Biophys Res Commun* **483**, 880-884 (2017). <https://doi.org:10.1016/j.bbrc.2017.01.027>

106	Tan, P. *et al.* SPOP inhibits mice pancreatic stellate cell activation by promoting FADD degradation in cerulein-induced chronic pancreatitis. *Exp Cell Res* **384**, 111606 (2019). <https://doi.org:10.1016/j.yexcr.2019.111606>

107	Luo, J. *et al.* SPOP promotes FADD degradation and inhibits NF-κB activity in non-small cell lung cancer. *Biochemical and biophysical research communications* **504**, 289-294 (2018). <https://doi.org:10.1016/j.bbrc.2018.08.176>

108	Liu, F. *et al.* Deficiency in SPOP-mediated ubiquitination and degradation of TIAM1 promotes gastric cancer progression. *Biochimica et biophysica acta. Molecular basis of disease* **1870**, 167032 (2024). <https://doi.org:10.1016/j.bbadis.2024.167032>

109	La, M. *et al.* Daxx-mediated transcriptional repression of MMP1 gene is reversed by SPOP. *Biochem Biophys Res Commun* **320**, 760-765 (2004). <https://doi.org:10.1016/j.bbrc.2004.06.022>

110	Zeng, C. *et al.* SPOP suppresses tumorigenesis by regulating Hedgehog/Gli2 signaling pathway in gastric cancer. *J Exp Clin Cancer Res* **33**, 75 (2014). <https://doi.org:10.1186/s13046-014-0075-8>

111	Song, Y. *et al.* The emerging role of SPOP protein in tumorigenesis and cancer therapy. *Mol Cancer* **19**, 2 (2020). <https://doi.org:10.1186/s12943-019-1124-x>

112	Wu, L. *et al.* [Speckle-type POZ protein up-regulates c-Jun protein expression and promotes proliferation and invasion of renal carcinoma cells]. *Nan fang yi ke da xue xue bao = Journal of Southern Medical University* **41**, 447-452 (2021). <https://doi.org:10.12122/j.issn.1673-4254.2021.03.19>

113	Zhao, W., Zhou, J., Deng, Z., Gao, Y. & Cheng, Y. SPOP promotes tumor progression via activation of β-catenin/TCF4 complex in clear cell renal cell carcinoma. *Int J Oncol* **49**, 1001-1008 (2016). <https://doi.org:10.3892/ijo.2016.3609>

114	Pang, K. *et al.* Degradation of DRAK1 by CUL3/SPOP E3 Ubiquitin ligase promotes tumor growth of paclitaxel-resistant cervical cancer cells. *Cell Death Dis* **13**, 169 (2022). <https://doi.org:10.1038/s41419-022-04619-w>

115	Li, R. *et al.* Effect of SPOP on Biological Behavior of Ovarian Cancer and Its Molecular Mechanism. *Genomics and Applied Biology* **39**, 1878-1885 (2020).

116	Jin, X. *et al.* CRL3-SPOP ubiquitin ligase complex suppresses the growth of diffuse large B-cell lymphoma by negatively regulating the MyD88/NF-κB signaling. *Leukemia* **34**, 1305-1314 (2020). <https://doi.org:10.1038/s41375-019-0661-z>

117	Li, Q. *et al.* SPOP promotes ubiquitination and degradation of MyD88 to suppress the innate immune response. *PLoS Pathog* **16**, e1008188 (2020). <https://doi.org:10.1371/journal.ppat.1008188>

118	Zang, L. *et al.* Ubiquitin E3 ligase SPOP is a host negative regulator of enterovirus 71-encoded 2A protease. *J Virol* **97**, e0078623 (2023). <https://doi.org:10.1128/jvi.00786-23>

119	Yu, G. Q. *et al.* Zebrafish spop promotes ubiquitination and degradation of mavs to suppress antiviral response via the lysosomal pathway. *International journal of biological macromolecules* **256**, 128451 (2024). <https://doi.org:10.1016/j.ijbiomac.2023.128451>

120	Pi, Y. *et al.* SPOP inhibits HBV transcription and replication by ubiquitination and degradation of HNF1α. *J Med Virol* **95**, e29254 (2023). <https://doi.org:10.1002/jmv.29254>

121	Wang, B. *et al.* Spop ameliorates diabetic nephropathy through restraining NLRP3 inflammasome. *Biochem Biophys Res Commun* **594**, 131-138 (2022). <https://doi.org:10.1016/j.bbrc.2021.12.068>

122	Zhu, K. *et al.* SPOP-containing complex regulates SETD2 stability and H3K36me3-coupled alternative splicing. *Nucleic Acids Res* **45**, 92-105 (2017). <https://doi.org:10.1093/nar/gkw814>

123	Takahashi, I., Kameoka, Y. & Hashimoto, K. MacroH2A1.2 binds the nuclear protein Spop. *Biochimica et biophysica acta* **1591**, 63-68 (2002). <https://doi.org:10.1016/s0167-4889(02)00249-5>

124	Hernández-Muñoz, I. *et al.* Stable X chromosome inactivation involves the PRC1 Polycomb complex and requires histone MACROH2A1 and the CULLIN3/SPOP ubiquitin E3 ligase. *Proceedings of the National Academy of Sciences of the United States of America* **102**, 7635-7640 (2005). <https://doi.org:10.1073/pnas.0408918102>

125	Wang, H. *et al.* Microglial TLR4-induced TAK1 phosphorylation and NLRP3 activation mediates neuroinflammation and contributes to chronic morphine-induced antinociceptive tolerance. *Pharmacological Research* **165** (2021). <https://doi.org:10.1016/j.phrs.2021.105482>

126	Sanada, S. *et al.* SPOP is essential for DNA replication licensing through maintaining translation of CDT1 and CDC6 in HaCaT cells. *Biochem Biophys Res Commun* **651**, 30-38 (2023). <https://doi.org:10.1016/j.bbrc.2023.02.012>

127	Cai, H. & Liu, A. Spop promotes skeletal development and homeostasis by positively regulating Ihh signaling. *Proc Natl Acad Sci U S A* **113**, 14751-14756 (2016). <https://doi.org:10.1073/pnas.1612520114>

128	Zhu, H. *et al.* SPOP E3 Ubiquitin Ligase Adaptor Promotes Cellular Senescence by Degrading the SENP7 deSUMOylase. *Cell reports* **13**, 1183-1193 (2015). <https://doi.org:10.1016/j.celrep.2015.09.083>

129	Jin, X. *et al.* Dysregulation of INF2-mediated mitochondrial fission in SPOP-mutated prostate cancer. *PLoS Genet* **13**, e1006748 (2017). <https://doi.org:10.1371/journal.pgen.1006748>

130	Ong, J. Y. & Torres, J. Z. Cul3 substrate adaptor SPOP targets Nup153 for degradation. *bioRxiv* (2023). <https://doi.org:10.1101/2023.05.13.540659>

131	Ostertag, M. S., Messias, A. C., Sattler, M. & Popowicz, G. M. The Structure of the SPOP-Pdx1 Interface Reveals Insights into the Phosphorylation-Dependent Binding Regulation. *Structure* **27**, 327-334.e323 (2019). <https://doi.org:10.1016/j.str.2018.10.005>

132	Liu, A., Desai, B. M. & Stoffers, D. A. Identification of PCIF1, a POZ domain protein that inhibits PDX-1 (MODY4) transcriptional activity. *Mol Cell Biol* **24**, 4372-4383 (2004). <https://doi.org:10.1128/mcb.24.10.4372-4383.2004>

133	Yang, W. *et al.* Substrate-dependent interaction of SPOP and RACK1 aggravates cardiac fibrosis following myocardial infarction. *Cell Chem Biol* **30**, 1248-1260.e1244 (2023). <https://doi.org:10.1016/j.chembiol.2023.06.015>

134	Lan, X. *et al.* The E3 ligase adaptor molecule SPOP regulates fetal hemoglobin levels in adult erythroid cells. *Blood Adv* **3**, 1586-1597 (2019). <https://doi.org:10.1182/bloodadvances.2019032318>
